# Early-career factors largely determine the future impact of prominent researchers: evidence across eight scientific fields

**DOI:** 10.1038/s41598-023-46050-x

**Published:** 2023-11-01

**Authors:** Alexander Krauss, Lluís Danús, Marta Sales-Pardo

**Affiliations:** 1 London School of Economics, London, UK; 2 Institute for Economic Analysis, Spanish National Research Council, Barcelona, Spain; 3 Department of Chemical Engineering, Universitat Rovira i Virgili, Tarragona, Spain

**Keywords:** Statistical physics, Scientific data

## Abstract

Can we help predict the future impact of researchers using early-career factors? We analyze early-career factors of the world’s 100 most prominent researchers across 8 scientific fields and identify four key drivers in researchers’ initial career: working at a top 25 ranked university, publishing a paper in a top 5 ranked journal, publishing most papers in top quartile (high-impact) journals and co-authoring with other prominent researchers in their field. We find that over 95% of prominent researchers across multiple fields had at least one of these four features in the first 5 years of their career. We find that the most prominent scientists who had an early career advantage in terms of citations and h-index are more likely to have had all four features, and that this advantage persists throughout their career after 10, 15 and 20 years. Our findings show that these few early-career factors help predict researchers’ impact later in their careers. Our research thus points to the need to enhance fairness and career mobility among scientists who have not had a jump start early on.

## Introduction

What drives high-impact science and how do scientists gain prominence? Can we help predict scientific success and especially the success of young researchers? And what would be the best metrics to do so? These are important questions in the science of science but that we still do not fully understand^1–9^. These questions are of interest for hiring committees, funding bodies and university departments who make decisions by trying to predict the scientific trajectories of researchers often using limited information. The use of common bibliometric indicators, such as number of publications, journal impact factors and citations, as metrics for assessing research impact has been put into question by some researchers^10,11^. Other metrics such as open access publications and altmetrics have been proposed as complements or alternatives for improving the way we assess research^10–12^. Yet any measure of scientific impact and prominence faces constraints. A necessary step in identifying ways to evaluate research more fairly is to apply predictive models that help identify inherent biases to science’s current incentive and evaluation system. To this end, we comprehensively analyze the careers of prominent scientists to identify to what extent early-career factors help predict the success of researchers later on in their career.

**Figure 1:**
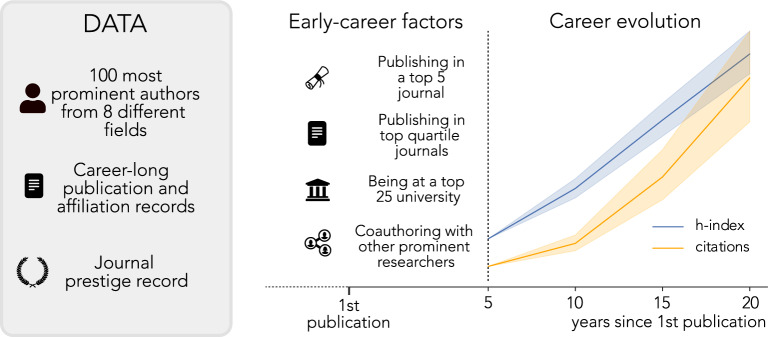
Conceptual map of the study. We compiled a list of the 800 most prominent scientists across 8 research fields. We obtained for each researcher a full publication list, history of citations of the publications as well as their affiliation records over time from Scopus. Using this information, we obtained data on early-career factors (within the first 5 years after their first publication): being at a top 25 university, publishing in a top 5 journal or most papers in Q1 journals within a specific area of knowledge (according to Journal Citation Reports), and coauthoring with other prominent researchers. We then study the subsequent career of the researchers and measure the evolution of their number of citations and h-index over 5, 10, 15 and 20 years since their first publication.

Most studies on the drivers of high-impact science focus on the role of an individual factor in isolation, such as the prestige and ranking of researchers’ university^13–16^, ranking of published papers in journals^17–19^, and collaborations^20–29^. Total citation counts and h-index of the world’s prominent scientists capture only past accomplishments, but not what has driven those achievements. Rarely are there studies conducted to identify the factors driving the production of high-impact research over time^7,8,27,30,31^, combining the different key factors in a single study to understand the relative importance of each factor^13–18^ and studying fields across the natural, behavioural and social sciences simultaneously^6,28,29^. Here, we do so by conducting a comparative analysis of these key factors to shed light on how early-career choices and factors shape the path to later become prominent researchers. To this end, we collected data on the scientific careers of the 100 most prominent scientists in eight different fields across science (genetics, development economics, cognitive psychology, network science, social inequalities in public health, network ecology, metabolomics, and philosophy of science) to which we apply a set of descriptive statistics, as well as classification and regression analyses (Data and Methods sections). Specifically, we examine four key early-career factors (researchers’ university prestige, journal ranking of their top publication, collaboration with other prominent researchers, and overall impact of their early research) which we find capture the scientific achievements during the first 5 years of the career. We then assess how these key factors are related to their h-index later on in their career, while controlling for factors like their geographic location^32–34^, gender^35^ and scientific field^23,35^ (Fig. 1).

We find that top researchers across fields have, in the first five years of their career, an advantage compared to the average researchers – the comparison group – that lasts throughout the rest of their career: they are more likely to research at one of the top 25 ranked universities worldwide, publish a paper in a top 5 ranked journal in their field, publish most papers in top quartile journals, and collaborate with other prominent researchers. Indeed, this trend holds for prominent researchers across scientific fields: the prominent researchers at the top of their field early on in their career (compared to their peers) are consistently at the top as their career progresses. Our results highlight how an *early-career jump-start* drives researchers to prominence, i.e. what a researcher does early on has a very strong impact on how they will perform in the future. The implications of our findings are vast and can provide young researchers with a means to evaluate their own expected career trajectories. Yet because these four attributes of ultra-successful scientists are predictable, the findings also suggest how closed the scientific system already is. The results also point to shortcomings in using the common and highly-influential indicators of success, namely citation and h-index metrics. This is because early career advantages–measured using these metrics–are so strong that they predefine ‘highly-successful scientists’ without further information about the content or social and policy impact of their research.

## Data

We collected data for several early-career factors, by building on a dataset we previously compiled that identified the 100 researchers with the highest h-index across eight fields that span across the natural, behavioural and social sciences (see^33^). These eight fields include genetics, development economics, cognitive psychology, network science, social inequalities in public health, network ecology, metabolomics, and philosophy of science. We extracted all data for this study - publications, bibliometric data, university affiliations etc. for each author - using Scopus database in 2021 (the largest database of peer-reviewed journals), with two exceptions–data for university rankings using QS World University Rankings 2021^36^ and for journal rankings using Journal Citation Reports (JCR) 2021^37^. All data used are publicly available via Scopus, QS World University Rankings and Journal Citation Reports (JCR).

To overcome shortcomings of studies with cross-sectional research designs (with data collected at one specific timepoint) we adopt a longitudinal research design by collecting data over the entire scientific career of the 100 prominent researchers across these fields. We use the h-index as a metric for prominence as it is designed to capture the quantity and quality of researchers’ output^38^. For each researcher we set the start of their academic career as the year of their first publication. We then collect data for the first 5 years of researchers’ scientific career, including their early-career university ranking, publication records and journal ranking, and collaborations. Table 1 provides a list of all main variables we study and the descriptive statistics for the variables that are disaggregated by scientific field. Some of these variables we collected are highly correlated, so they were discarded for the analysis we later perform (see Supplementary Figs. S26).

All data presented throughout the paper reflect only the first 5 years of prominent researchers’ careers since their first publication, unless explicitly stated otherwise - i.e. with the exception of the number of accumulated citations and the h-index at 10, 15 and 20 years after the first publication of each scientist. All data are presented at the researcher level, i.e. only one aggregate value for each of the 100 prominent researchers across eight fields is provided for each variable. The 100 prominent researchers across these 8 fields have an average h-index of 64, meaning that researchers each have an average of 64 publications that have each received at least 64 citations. The median h-index is 49. In contrast, the average global h-index is approximated at 27 - 32 (median 14–25) as an upper bound estimate (see Table 1 for field-level data)^39,40^.

Moreover, as nearly all of today’s prominent researchers were based in Europe and North America in the first five years of their career and to allow for cross-regional comparison, we focus the analysis on Europe and North America—excluding about 6% of other prominent researchers not based there. Among the prominent researchers across each of the eight fields, 21 researchers were at a university outside of Europe or North America at the time of their first publication (largely in Australia, New Zealand and Japan), while most moved within the first five years to a university in Europe or North America to which they have been classified.

**Table 1:** Descriptive statistics. Features and traits of the 100 prominent researchers across each of the eight fields.

Average for the 100 prominent researchers in each field (all data reflect the average per researcher in the given field, unless stated otherwise)	Gene.	Dev. Eco.	Cog. Psy.	Net. Sci.	Ineq. Hea.	Net. Eco.	Metab.	Phi. Sci.	All fields
Total H-index – mean	130	62	89	43	63	43	48	31	64
Total H-index – median	136	53	86	37	56	41	41	25	49
H-index at 20 years since first publication – mean	35	16	14	30	27	23	29	7,2	23
H-index at 20 years since first publication – median	29	14	12	27	24	22	28	6	19
First 5 years of career									
% at one of the top 25 ranked universities worldwide in first 5 years	0,56	0,71	0,67	0,34	0,38	0,28	0,37	0,45	0,47
% who published a paper in a top 5 ranked journal in their field in first 5 years	0,93	0,74	0,82	0,79	0,74	0,77	0,86	0,51	0,77
% of researchers’ total papers in top decile (journal rankings) in first 5 years	0,42	0,26	0,28	0,27	0,27	0,34	0,33	0,09	0,27
% of researchers’ total papers in first quartile (journal rankings) in first 5 years	0,73	0,51	0,66	0,60	0,50	0,61	0,71	0,38	0,59
% with more than half of researchers’ total papers published in first quartile journals in first 5 years	0,83	0,56	0,77	0,69	0,55	0,73	0,87	0,38	0,68
% who co-authored a paper in first 5 years with another prominent 100 researcher in their field	0,26	0,31	0,28	0,42	0,25	0,27	0,24	0,11	0,27
% of researchers’ total papers in first 5 years that are coauthored with another prominent 100 researcher in their field	0,09	0,11	0,09	0,19	0,13	0,13	0,13	0,03	0,11
Total citations in first 5 years	137	23	14	97	33	35	61	8	52
Average number of authors for researchers’ total papers in first 5 years	16,4	1,7	1,8	10,3	3,9	3,6	5,3	1,8	5,9
% of researchers’ multi-author papers (among all their papers) in first 5 years	0,88	0,59	0,59	0,87	0,79	0,78	0,97	0,28	0,72
Average Journal Impact Factor for researchers’ papers in first 5 years	82,9	70,7	75,8	74,8	67,6	75,7	79,6	60,1	73,6
% at North American university at their first publication	0,66	0,69	0,74	0,41	0,47	0,39	0,38	0,66	0,55
% at EU university at their first publication	0,34	0,31	0,26	0,59	0,53	0,61	0,62	0,34	0,45
Present researcher status (early 2021)									
% presently at North American university/institution	0,75	0,72	0,81	0,55	0,48	0,44	0,43	0,73	0,62
% presently at EU university/institution	0,25	0,28	0,19	0,45	0,52	0,56	0,57	0,27	0,38
% male	0,94	0,87	0,85	0,86	0,71	0,86	0,81	0,86	0,85

## Results

### Four early-career factors related to early-on prominence and research impact

#### Early-career factors of prominent scientists

We analyse the first 5 years of the academic career (starting at the first publication) of the 100 prominent researchers across these eight scientific fields, and we find overall that 47% were at a top 25 ranked university, 77% published a paper in a top 5 ranked journal in their field, 59% of their papers were published in top quartile (Q1) journals and 27% co-authored a paper with another prominent researcher in their field (Table 1). These shares are significantly higher than for the comparison group of average researchers (see Methods for calculations for the global averages for researchers and Table 1 for all factors we analyzed): less than 1% of all researchers worldwide—an estimated 0.6%—are at one of the top 25 universities; an estimated 3–14% of all researchers worldwide have published a paper ranked in the top 5 journals in their field; about one third of all articles worldwide are published in top quartile (Q1) journals;^41,42^ and, about 14% of junior researchers on average have co-authored a paper with a senior researcher in journals across scientific fields, including top multidisciplinary journals.

**Figure 2:**
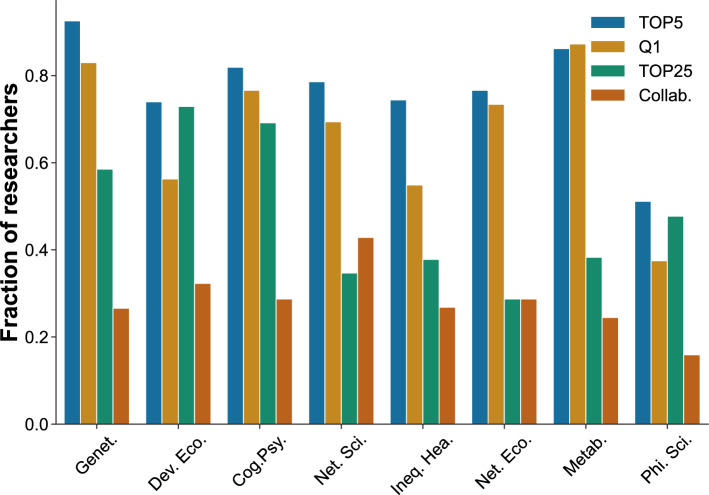
Early-career factors of prominent researchers across fields. Fraction of researchers by field for the four key variables in the first 5 years since the first publication: TOP5 represents whether a researcher published in a top 5 ranked journal in their field. Q1 represents whether a researcher published most of their papers in a top quartile journal. TOP25 represents whether a researcher was affiliated to one of the top 25 universities worldwide. Collab represents whether a researcher co-authored a paper with another prominent researcher in their field.

Furthemore, 92% of all prominent researchers had at least one or more of these four features, with the share increasing to at least 95% for those in genetics, development economics, cognitive psychology and metabolomics. Moreover, more than half of all prominent researchers placed a paper within a top 5 ranked journal in their field in the first 5 years, with the highest shares at 93% for researchers in genetics, 86% in metabolomics and 82% in cognitive psychology (Fig. 2). The majority of prominent researchers publish more than half of their papers in top quartile journals (except for philosophy of science) (Fig. 2). As we will show later, this initial prominence is often not just a ‘hot streak’ but consistently characterises the impact of researchers’ over their career.

A researcher’s early institution is also strongly correlated with scientific prominence across a number of fields^13–16^. Indeed, we find that over 50% of researchers in development economics, cognitive psychology, and genetics were at one of the top 25 ranked universities worldwide in the first 5 years of their career. However, this is not the case in younger scientific fields such as network science, network ecology or metabolomics, suggesting that the role of institutional prominence seems to be more important in well-established, more traditional fields. Being at a top university is the factor, among the four early-career factors, that illustrates the strongest difference between newer and older fields. Another factor that highlights differences between fields is the collaboration network that prominent researchers establish. Network science is the most collaborative field (in which 42% of prominent researchers have co-authored a publication with another prominent researcher) while philosophy of science stands out as the least collaborative (in which 17% of prominent researchers have done so) (Fig. 2).

In terms of geographic differences, we find that prominent European researchers are, in their early career, overall more likely to have top publications and to have been at a top 25 ranked university across all fields (Supplementary Fig. S1), even though North America has a larger concentration of top universities whose graduates occupy the majority of faculty positions in US universities^43^. Prominent European researchers are, however, less likely to have co-authored a paper with another of these top 100 researchers in their field, except in development economics and cognitive psychology (Supplementary Fig. S1)^33^.

In terms of gender differences, our results confirm that the gender gap is even more exacerbated among the scientific elite: females account for 15% of all prominent researchers across fields, ranging from 29% in inequalities in public health to only 6% in genetics^35^. In the first five years, prominent female researchers have a similar (or even higher) share of papers in the top quartile as males across fields, except in genetics. They are also more likely to have researched at a top 25 university than males across fields, except in network science and philosophy of science, and a larger fraction of women has also coauthored a paper with another prominent researcher (Supplementary Fig. S2).

#### Early-career factors are correlated with early-on research output

**Figure 3:**
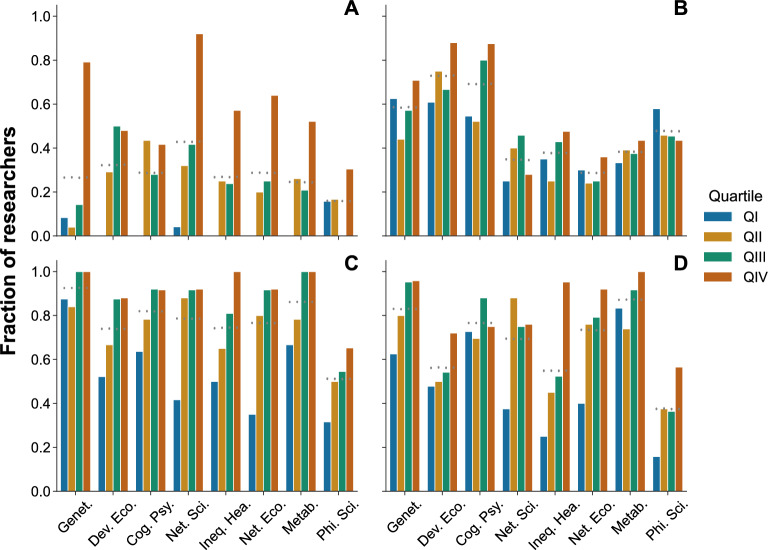
Early-career factors of prominent researchers disaggregated by citation quartiles. Fraction of researchers by field and quartile in the first five years who have: (**A**) Publications with other prominent researchers in their field. (**B**) Affiliation in one of the top 25 universities. (**C**) A paper published in a top 5 ranked journal in their field. (**D**) Most of their papers published in a top quartile journal in their field. Grey points represent the values and the 95% confidence interval expected when radomizing the citation quartiles within each field.

To understand the relationship of early-career factors to early performance, we disaggregate researchers into four quartiles of increasing number of citations they received during the first five years (i.e. researchers in quartile 1 (QI) are those with the lowest 25% of citations received during the first five years, while researchers in quartile 4 (QIV) – the top cited quartile – are those with the highest 25% of citations). We find that there is a strong correlation between the four early-career drivers and the impact of research output early on in researchers’ career. The fraction of prominent researchers in the top citation quartile in the first five years are, in general, more likely than expected by chance to have any of the four early-career features than other prominent researchers in lower citation quartiles (Fig. 3).

#### The role of publishing with other prominent researchers

Collaboration among scientists has been recognised as a source for innovation and creativity leading to increased research output^20,21^. Our analysis is consistent with these findings: co-authorship is strongly correlated with higher citations across all fields, and the relationship is particularly strong in the natural sciences including genetics and network science (Supplementary Fig. S3).

Remarkably, the effect of co-authoring with prominent researchers is even greater. We find that only 27% of prominent researchers co-authored at least one paper (and overall 11% of their papers) with another prominent researcher in the first 5 years of their career. The papers co-authored by two (or more) prominent researchers have a much higher number of citations than other papers. The effect, intensity and size of collaborations, however, is not homogeneous across geographic locations^33^ nor across fields (Supplementary Fig. S1, S4D and S5). Furthermore, the disaggregated data by citation quartiles reveal that researchers in the lowest citation quartile have very low shares of co-authorship in their early career across fields with other prominent researchers in their field compared to an average of 56% for those in the top citation quartile (Fig.  3A). This finding suggests that co-authorship with other prominent researchers early on can have a large return across all fields. Indeed, already during the first five years of the career of scientists in our study, papers with other prominent scientists have overall received more than twice the number of citations than those not co-authored with other prominent scientists in their field (Supplementary Figs. S4D and S5).

Our findings are thus in line with previous studies that analyzed the advantages of co-authoring with leading researchers in one’s field. Working under leading researchers can boost career development through greater citations and mentorship^44^, and provides visibility early on in a scientist’s career^26^. In fact, junior scientists at less recognised universities are most likely to benefit from co-authorship with leading researchers^26^. Young scientists can also apply what they learn from high-impact, established researchers in their own career^27,28,45^, providing them with a competitive advantage relative to their peers^46^.

#### The role of prestige of researchers’ institution

Researchers at top universities have a qualitative advantage with respect to researchers in other institutions. They enjoy a high-quality research environment, generally with access to greater resources. Additionally, researchers at prestigious institutions are sought for collaboration as a way to boost the academic careers of researchers at lower tier institutions^22^. Here, we assess the relationship between being at a top university and early-career impact. The share of researchers who have spent part of their early career in such institutions is not homogeneous across fields, with traditional disciplines having much larger shares, as outlined earlier. Not surprisingly, we find that for these disciplines – genetics, development economics and cognitive psychology – being at a top university is strongly correlated with early-on research impact. Nonetheless, across most fields we find that researchers most cited early on in their career are more likely to be in a top institution (Fig.  3B; Supplementary Fig. S4C).

Researchers at prestigious universities also have a comparative advantage on other indicators. Among these prominent researchers at a top 25 university in the first 5 years of their career, 79% published a paper in a top 5 journal (compared to 76% at a non-top university), 72% published more than half of all papers in top quartile journals (compared to 64%) and 29% co-authored with another prominent researcher (compared to 25%) (Supplementary Fig. S6).

#### The role of publishing in highly-ranked journals

Publishing in high impact journals early on is correlated with an increase in later impact – by increasing citations it benefits researchers’ career opportunities, increases their prestige and recognition, and helps promotion^18^. Nearly all prominent researchers across fields placed their best paper in their early career within a highly ranked journal, which thus appears to be a necessary condition for becoming a prominent researcher. In fact, publishing in highly-ranked journals is strongly correlated with greater early-career impact, more so than just publishing within journals in Q1 (Fig.  3C, D). Interestingly, these two early-career factors (publishing in a top 5 journal, versus publishing the majority of articles in Q1 journals) are not highly correlated with each other (Supplementary Fig. S26), thus showing that these two variables characerise two different aspects of early career performance: the former characterises the big hits, while the later represents consistency in output quality, and therefore are distinct early-career factors.

### Early-career performance is a strong indicator of performance throughout later career stages

As the scientific career of researchers progresses, the number of publications and citations accrued increases and so does the h-index of each researcher (Supplementary Fig. S7). We find disparities between fields in terms of the evolution of h-indices over time which reflect differences in the rate of publications, collaboration structures and the size of each field (Supplementary Fig. S8) .

**Figure 4:**
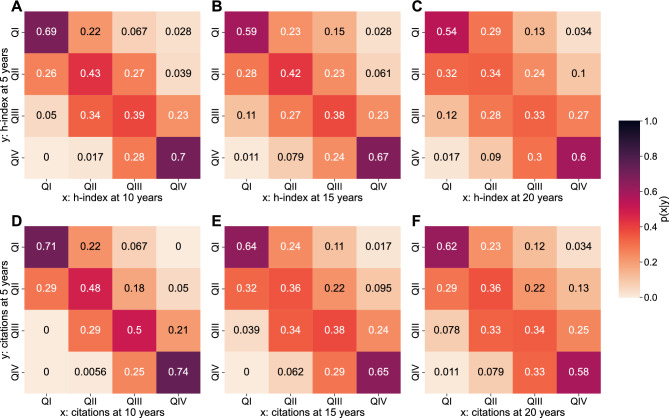
Researcher mobility across quartiles. H-index quartile at five years compared to h-index quartile at 10, 15 and 20 years (first row, panels **A**–**C**), and citation quartile at five years compared to citation quartile at 10, 15 and 20 years (second row, panels **D**–**F**). The darker the region, the stronger the coincidence between the quartile at 10, 15 and 20 years relative to the quartile at the first 5 years. The results reflect the aggregated and normalized data for all fields.

To assess whether early-career performance translates into a sustained advantage over time, we analyse the evolution of h-indices and citations over time for all researchers (pooled together across the eight fields) (Fig. 4). To this end, we divide researchers into quartiles based on the normalized h-index and the normalized number of citations at 5, 10, 15 and 20 years since the first publication (see Methods). We then look at the probability of transition over time between quartiles using the 5-year mark as the reference point (Fig. 4). We observe that the initial advantage in the first 5 years is still present at 20 years of researchers’ career. Figure 4C and F shows that 90% of researchers that started their career in the two top citation quartiles (QIII and QIV) have maintained this prominent position over time. Conversely, we observe the same situation for those scientists who were in the lower two quartiles (QI and QII). Both findings are consistent, whether we look at quartiles defined by h-index (Fig. 4 first row) or by citations (Fig. 4 second row) and across fields (Supplementary Figs. S9–S10). Although some fields display greater mobility from lower to upper quartiles, such as in network science and metabolomics, researchers are very unlikely to transition from the top-two to the bottom-two quartiles. This suggests that the initial advantage consistently remains throughout researchers’ career.

### Factors driving citations and h-index in researchers’ early career

**Figure 5:**
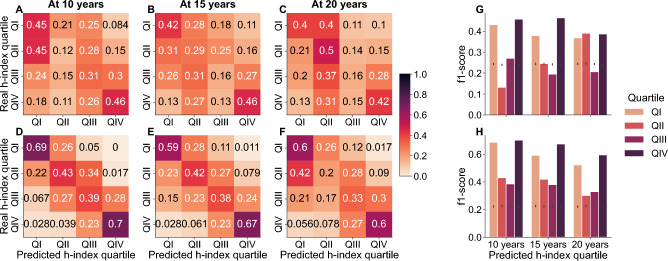
Prediction of h-index quartile based on early-career factors. Predicted h-index quartile at five years compared to observed h-index quartile at 10, 15 and 20 years (first, second and third columns). (**A**)–(**C**) illustrate the prediction results with Model 2 (which takes into account the four early-career factors as well as the geographic location and gender of researchers; see Methods). (**D**)–(**F**) illustrate the prediction results with Model Q5 (which only takes into account the quartile of the first 5 years). The darker the region, the higher the number of researchers that are correctly classified by the algorithm. The results reflect the aggregated and normalized data for all fields. (**G**) and (**H**) show the f1-score for the predictions of Model 2 and Model Q5, respectively. Grey bars represent the 95% confidence interval expected when predicting randomized citation quartiles.

So far our results in Figs. 3 and  4 show that there is a clear relationship between key early-career factors and the early-on impact of research output, and between early-on impact of research output and impact at later career stages. Here, we want to assess the extent to which early-career factors can explain the evolution in the impact of research output during the career of prominent scientists. To this end, we perform a prediction experiment in which we consider the h-index/citation quartile of a researcher (which we obtain by pooling together normalized h-indices of the 8 fields) at Y (=10, 15 or 20) years after the start of their scientific career as our dependent variable, and different combinations of early-career factors as well as researchers attributes such as gender or current geographic location as our independent variables (see Supplementary Fig. S26 for evidence of lack of co-linearity among independent variables). Specifically, we train a Random Forest classifier for different sets of independent variables, called *Models* (see Methods for a description of the models—Model 1, 2, 3 and Q5) (Fig. 5). In Fig.  5, we show the prediction results of two classifiers for two different Models (sets of independent variables). First, we train a classifier in which we use binary independent variables that account for the four key early-career factors we study—working at a top 25 ranked university, publishing a paper in a top 5 ranked journal, publishing most papers in top quartile journals, and co-authoring with other prominent researchers—as well as two common background factors, namely researchers’ geographic location and their gender, called Model 2 (see Methods and Supplementary Material for other models we analyse). Second, we train a classifier in which the only independent varible we considere is the h-index quartile after 5 years of the first publication (Model Q5).

Our classification analysis reveals that assessing the h-index quartile at 5 years (Model Q5), the classifier is more accurate than if we only include the early-career factors. Nonetheless, the classifier for Model 2 is still able to correctly predict overall 40% of the researchers that fall into the lowest quartile (QI) and 38% who fall into the top quartile (QIV) at 20 years from the start of their career—significantly higher than the expected 25% for random quartile assignment. Our results show that the early-career factors we study can explain h-index quartiles as early as 5 years after the first publication (Supplementary Figs. S11–S12) as well as trends in the share of researchers who remain in the same h-index quartile over their career (Fig.  5; see also Figs. S13–S14 for an equivalent analysis for citations). We also observe that for both classifiers, missclassification tends to happen between neighboring quartiles, so that the fraction of lower quartile researchers are seldom classified as QIV researchers and vice versa. Indeed, f1 scores highlight precisely that in Model2 and Q5 the performance for Q1 and QIV is better than for QII and QIII (Fig. 5 H and G; see Supplementary Fig. S15 for precision and recall for the same models). This indicates that early-career features (Model 2) capture a substantial part (but not all) of the information captured by the h-index (Model Q5). Nonetheless, our results show that early-career researchers who are already prominent among their peers are very likely to sustain their advantage 15-20 years later (i.e. researchers in QIV). We find consistent results when we analyse fields individually (Supplementary Figs. S16–S19 for h-index quartile prediction; Supplementary Figs. S21–S24 for citation quartile prediction).

As a final step, using our trained Random Forest classifiers for Model 2, we analyze the relative importance of the key four early-career factors (Methods). As all variables are binary (0 or 1), this facilitates comparing the relative importance of each factor. Collaborating with other prominent researchers is the most important factor, followed by publishing a paper in a top 5 journal. Working at a top 25 university and publishing more than half of one’s papers in Q1 journals have less explanatory power; and gender and geographic location appear to have little predictive power (Supplementary Figs. S14, S20, S25). The results illustrate how collaborating with established researchers is perhaps the best strategy for securing a position among the scientific elite. These results are consistent with results from the analysis of citations (Supplementary Fig. S20) and with the disaggregated analysis of individual fields (Supplementary Figs. S20, S25). The only exception is philosophy of science for which being at a top institution or publishing in top journals early on are better predictors of h-index and citation quartiles while publishing with other prominent scientists is of much less importance. As a final robustness check, we perform two different regression analyses: an ordinary least squares regression of the h-indices, and a logistic regression of the top tercile of h-indices (see Figs. S27, S28, and Supplementary Table S1), which confirm the relative importance of variables we obtain using the Random Forest classifier.

## Discussion

Our analysis shows that the future success of a researcher is often determined early on in their career. Indeed, we show that as early as 5 years after the first publication, we can already make accurate predictions of whether a prominent researcher is going to be within the top quartile of leading researchers later on or not. Our study, while limited to prominent scientists, shows that early-career factors also establish a hierarchy within this group of scientists that is sustained over time.

We find four early-career factors that are central drivers for later success across science: working at a highly ranked university, publishing a top 5 journal paper, publishing most papers in top quartile journals and co-authoring with prominent researchers at the early stage of researchers’ career. Most importantly, we find a strong positive correlation between citations during the first five years of their career and the probability to have had any of these central early-career features we identify: researchers in the top quartile of citations are more likely than expected to have the four key features, whereas researchers in the lowest citation quartile are less likely than expected to have these features (but still more likely than the average non-prominent researchers). This finding is very insightful, especially because classification models are able to accurately predict the citation and h-index quartiles after 10, 15 and 20 years for researchers falling into the top and lowest quartiles: what scientists do early on largely determines their impact later on in their careers.

We also find that in traditional areas of science, being at a top-ranked institution can be an important driver, but in younger disciplines it is less important. This finding is especially interesting in light of recent findings about graduates from top-ranked US universities occupying the majority of faculty positions in the US university ecosystem^43^, and raises the question of whether hierarchies in the hiring system pose a threat to innovation and the emergence of new fields of science. Indeed, we also find that in disciplines in which university affiliation is not such an important driver, publishing with other prominent scientists becomes especially important^44^.

Our analysis shows that these four key factors are important as a general strategy for young researchers across science and that an early-career jump start gives scientists an advantage that is sustained throughout their career. At the same time, our results suggest that there are also other factors influencing the h-index at 5 years such as individual, more qualitative or psychological traits of researchers^19^ or, in relevant cases, the traits of a PhD advisor^45^ that have not been considered here. While it can be a limitation, our results also explain that the success of individual researchers cannot be attributed to a single factor but involve a combined set of early-career factors.

Given that these four attributes of ultra-successful scientists are predictable, the findings suggest that the scientific system is presently relatively closed. The results also illustrate limitations of using highly-influential metrics of success, such as citations and h-index. This is because early career advantages on these metrics are so strong that they predefine ‘highly-prominent scientists’, independent of the content of their research. More generally, the findings point to the need for a reform among the scientific community: As some scientists produce good science but are not successful in the ‘metrics game’, decision makers evaluating the work of researchers should also use additional metrics such as policy and social impact of research, developing new research tools, and the like. Decision makers should thus by no means take this as an opportunity to just use citation and h-index metrics to evaluate scientific prominence.

Overall, our *jump-start hypothesis* here can, by integrating multiple early-career factors and not focusing on an individual factor in isolation, better explain the Matthew effect in science^47^, namely how the most cited researchers get more cited just because they became highly cited early on in their career. The central implication for researchers is that early-career factors can be fostered through deliberate choices and hard work.

## Methods

### Calculations for the average researchers globally (the comparison group)

The calculations for the average researchers globally—the comparison group—for the four factors analysed here have been made as follows. Firstly, less than 1% of all researchers worldwide—an estimated 0.6%—are at one of the top 25 universities. This share is calculated using UNESCO data on the total number of researchers worldwide at 8,854,288^48^ divided by the total number of researchers (university staff) at the same top 25 universities (using QS World University Rankings) at 56,900. For comparison, the top 25 universities account for 1.8% of the total 1396 universities in the Times World University Rankings^49^. Secondly, an estimated 3−14% of all researchers worldwide have published a paper ranked in the top 5% in their field. This share is calculated by using data on the total number of all publications ranked top 5% in researchers’ field at 267,966 publications indexed in Web of Science using the Leiden Ranking^50^ divided by the total number of researchers worldwide at 8,854,288^48^ or by the total number of researchers (university staff) at 1,914,149^49^ that results in a 3% (lower bound) or 14% (upper bound) estimate, respectively. Thirdly, about one third of all articles worldwide (upper bound estimate) are published in top quartile journals indexed in Web of Science;^41,42^ and as many individual researchers publish multiple articles in quartile 1 journals it is likely that the share is significantly lower for the average researchers to publish at least half of their papers in quartile 1 journals. Fourthly, about 14% of junior researchers on average have co-authored a paper with a senior researcher between 1990 and 2012 in a global study covering about 1000 journals across the sciences (totalling about 6 million publications), with the shares varying across the fields of biology (15%), physics (13%), chemistry (13%), medicine (16%) and mathematics (6%), including the top three multidisciplinary journals (Nature, Science and PNAS) at about 19% for each journal^44^. Fifth, the average h-index using university-level data is estimated at about 27 (median 25) as an upper bound estimate that includes only the top 500 universities^40^. The average h-index using all journal-level data from the Scimago Institutions Ranking^39^ via Scopus is estimated at about 32 (median 14). Note that both the mean university-level and journal-level h-indexes are upper bound estimates - i.e. higher than the mean researcher-level h-index given that researchers with lower h-indices are not represented in such estimates. These averages for researchers globally provide the baseline comparisons for our analysis.

### Statistical approaches and Models (sets of independent variables)

We use two statistical approaches, a Random Forest classifier and a linear regression, to understand the role that different early-career variables play in the evolution of the h-indices and accumulated citations over the duration of scientists’ career.

Our goal is to assess how well different factors help predict researchers’ h-index/citation counts (the dependent variables). We thereby consider four different groups of independent variables that we denote as Models 1, 2, 3 and Q5. Formally, we will refer to the sets of variables as $M_1, M_2, M_3, M_{{\rm q}5}.$

#### Models 1, 2 and 3

For these three Models, all independent variables are binary (0 or 1). Descriptive data for all variables used in the models are provided in Table 1. Supplementary Figure S26 shows that there is no strong correlation between the different variables we consider in what follows.

*Model 1*. This model considers as independent variables *solely* the four key early-career factors we study, namely working at a top 25 ranked university or not ($\textrm{topU}$)^13–16^ publishing a paper in a top 5 ranked journal or not ($\textrm{top5}$), publishing most papers in Q1 journals or not (*Q*1)^17–19^ and co-authoring with other top 100 researchers or not ($\textrm{BS}$)^23,26–29^. Therfore $M_1:=\{\textrm{TOP25},\textrm{TOP5},Q1,\mathrm{Collab.}\}$.

*Model 2*. This model considers the same variables as in Model 1 but also controls for two common background factors: the researchers’ geographic location ($\textrm{loc}$: whether they are based at a university in North America or not)^32–34^, and their gender ($\textrm{Gender}$: whether they are male or not)^35^. These are standard control variables applied in economics and the social sciences. Therefore, $M_2:=\{\textrm{TOP25},\mathrm{\mathrm TOP5},Q1,\mathrm{Collab.}, \textrm{Firstloc}, \textrm{Gender}\}$.

*Model 3*. This model considers the same variables as in Model 2 but also controls for the average number of co-authors on researchers’ total papers ($\textrm{coaut}$), so that $M_2:= \{\textrm{TOP25},\mathrm{\mathrm TOP5},Q1,\mathrm{Collab.}, \textrm{Firstloc}, \textrm{Gender}, \textrm{Avg}\}$).

*Model Q5*. This model considers only the h-index quartile at 5 years after first publication (*Q*5), $M_{Q5}:= \{Q5\}$.

#### Random forest classifier

In order to quantify the predictive power of the models and the different variables, we performed a classification experiment using a Random Forest Classifier. Our goal was to assess whether we could correctly predict the h-index/citation quartile at 5, 10, 15 and 20 years of career using only indicators from the first 5 years since the first publication.

A Random Forest Classifier (RFC) behaves similarly to a Random Forest Regressor but produces a categorical output instead of a continuous one. In this sense, the classifier iteratively evaluates several decision trees over different parts of the data and averages the resulting outputs.

We evaluated the performance of the classifier with a 10-fold cross validation. In this procedure, the dataset is divided in 10 folds from which one is selected as the test and the others as the training folds iterated several times until each fold has been used as a test. For each one of the models $M=\{M_1,M_2,M_3,M_{Q5}\}$, training data for each fold $F=\{\textrm{training}_F,\textrm{test}_F\}$ corresponds to $Tr_F (M):=\{ (QY_i, \textbf{x}_i), \,i \in \textrm{training}_F\}$ where $QY_i$ is the quartile at year *Y* we want to predict, and $\textbf{x}_i$ are the feature values or independent variable values $\textbf{x}_i=\{\left( M \right) _i\}$ for a specific model (and similarly for test data). For each $Tr_F(M)$, we train a Random Forest Classifier $\textrm{RFC}_{F,M}\equiv \textrm{RFC}(Tr_F(M))$ and make predictions for the corresponding test set $\{\widehat{QY}_j(M)=\textrm{RFC}_{F,M}(\textbf{x}_j), \,j \in \textrm{test}_F\}$. Since test sets are non-overlapping, in the end we obtain a list of $\{\widehat{QY}_j(M), \forall j\}$, which we compared to the real quartiles $\{QY_j, \, \forall j \}$ to obtain the overall confusion matrices, precision and recall for each model *M*. We then select the best model from Models 1, 2 and 3 as the one with the best overall precision and recall, in our case Model 2 ($M_2$).

Note that when performing the classification analysis for the aggregated data comprising all fields, the h-index and citation data are normalized due to high variability among fields (Supplementary Fig. S8).

*Feature importance.* For each $\textrm{RFC}_{F,M}$ we obtain permutation feature importances for each independent variable, that is, the feature importance of variable $\textrm{top5}$, $FI_{F,M}(\textrm{top5})$ is the reduction in performance of the Random Forest Classifier when we randomize $\textrm{top5}$.

Formally, the feature importance of a variable *v* is defined as:

$$\begin{aligned} FI_{F,M}(v) = s-\frac{1}{K}\sum _{k=1}^K s_{k,v}(D_{F/ v}) \end{aligned}$$

where $s_{k,j}$ is the score function, *s* the reference score, $D_{F/ j}$ the new dataset with variable *v* randomized, *k* is the repetition, and *K* the number of repetitions. To obtain the overall feature importance for a variable *v*, we average over folds $FI_{M}(v)= \frac{1}{10}\sum _{F=1}^{10} FI_{F,M}(v)$.

In our case, we selected the f1-score as the score function for the permutation importance and set K = 10 repetitions.

*f1-score, precision and recall.* The values of performance metrics for the RFC shown in Figure 3 and in Supplementary Fig. S15 are the results of averaging these metrics over folds in our classification analysis. Black bars in those figures show the 95% confidence interval when assessing the same metrics over a Random Forest Classifier trained with random assignation of quartiles to researchers.

#### Regression analysis

To assess the predictors of scientific prominence, we analyse which early-career factors influence an increase in citation counts most. We perform ordinary least squares (OLS) and logistic regression analyses.

The OLS results illustrate the mean change in the dependent variable (researchers’ h-index or their total citations in their early career) given a one-unit change in each independent variable (being at a top 25 university or not, being in North America or not etc.). All independent variables are binary (0 or 1). Specifically, the model is $y_i(M)=a_0+\sum _{i\in M} a_i x_i$, where the dependent variable *y* is the normalized h-index/number of citations and $\textbf{x}_i$ are the independent variables we consider in Model $M =\{M_1, M_2, M_3\}$.

We perform OLS regression analysis to assess the predictors of h-index in the first 5, 10, 15 and 20 years for the world’s prominent researchers (see Supplementary Fig. S27 and Supplementary Table S1 for regression coefficients and significance) and to predict the number of citations in the first 5 years (Supplementary Fig. S28B).

Second, we conduct a logistic regression analysis in which the binary dependent variable $y_i$ is equal to 1 for the third most-cited top researchers in the first 5 years and $y_i=0$ for the bottom two-thirds least cited top researchers. These top third researchers reflect the best of the best in their field. We thereby normalise citations by calculating citation terciles for each field individually (Supplementary Fig. S28 A). The model in this case corresponds to $p(y_i(M))= 1/\left[ 1+\exp \left( -f(M,\textbf{x}_i)\right) \right]$ where $f(M,\textbf{x}_i)=a_0+\sum _{i\in M} a_i x_i$. The coefficients $a_i$ thus express how the probability of $p(y=1)$ changes when $x_i=1$ (positive coefficients increase the probability, while negative ones decrease it); $a_0$ is a coefficient that sets the background probability for $p(y=1)$ – 1/3 in our case.

### Supplementary Information

Supplementary Information 1.

## Data Availability

All data are publicly available, and the lists of prominent researchers and their publications can be provided upon request (a.krauss@lse.ac.uk, marta.sales@urv.cat).
