# Supplementary material for: Early-career factors largely determine the future impact of prominent researchers: evidence across eight scientific fields
Source: Sci Rep. 2023 Nov 1;13:18794. doi: 10.1038/s41598-023-46050-x (PMC10620415; doi:10.1038/s41598-023-46050-x)
Supplement: Supplementary file 1 — Supplementary Information 1. [file 41598_2023_46050_MOESM1_ESM.docx]

**Supplementary Figures**

**Early-career factors largely determine the future impact of prominent researchers: Evidence across eight scientific fields**

Alexander Krauss^1,2^; Lluís Danús^3^; Marta Sales-Pardo^3^

^1^London School of Economics. ^2^Institute for Economic Analysis, Spanish National Research Council. ^3^Department of Chemical Engineering, Universitat Rovira i Virgili, Catalonia.


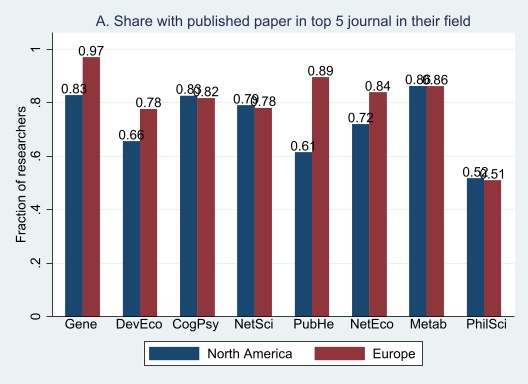

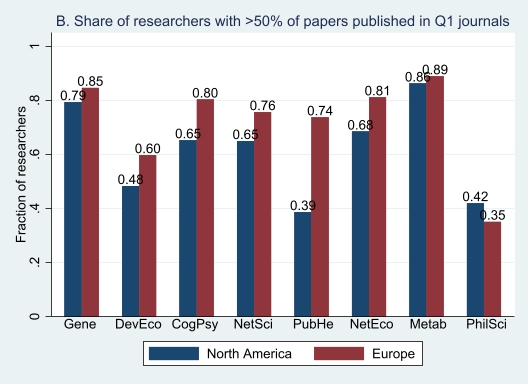


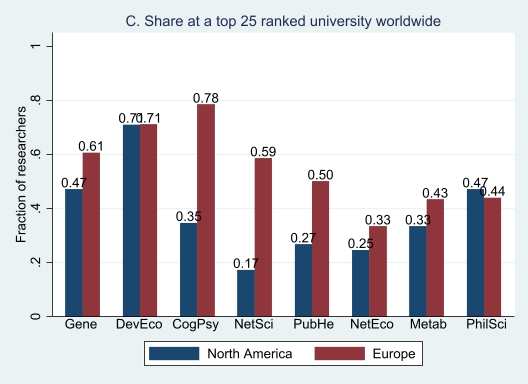

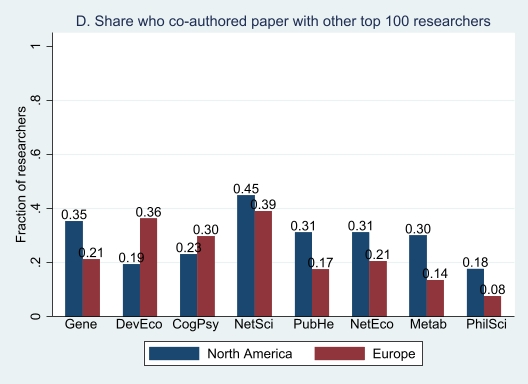


**Supplementary Figure S1. Early-career factors of the 100 prominent researchers across fields and geographic location in Europe and North America.** All data reflect the first 5 years of researchers’ career.

*
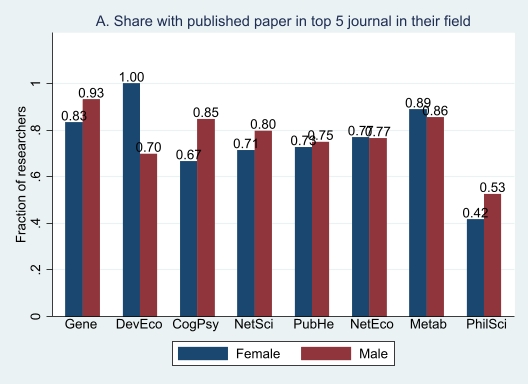

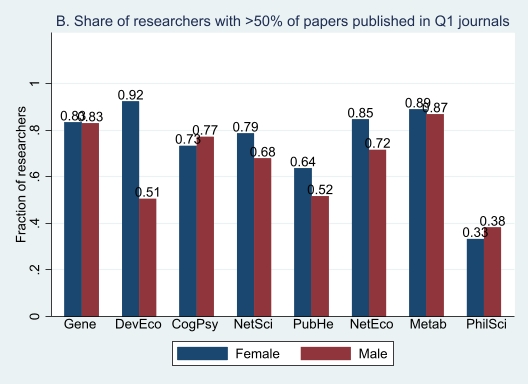

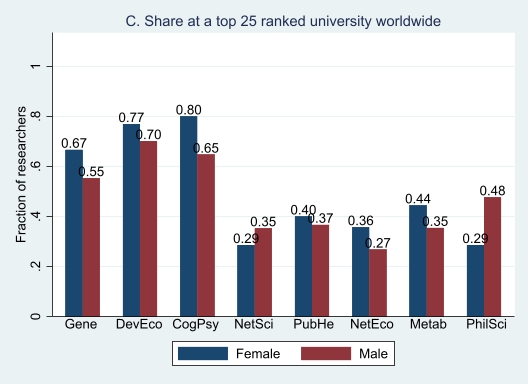

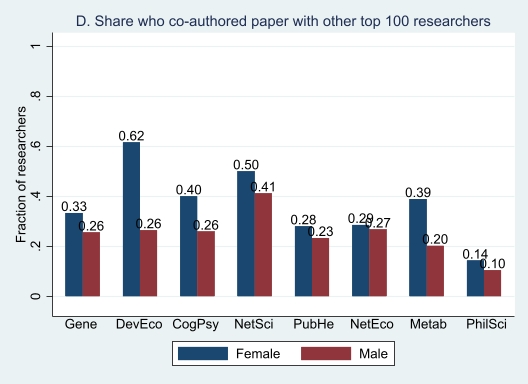
*

**Supplementary Figure S2. Early-career factors of the 100 prominent researchers across fields and gender.** All data reflect the first 5 years of researchers’ careers.

**
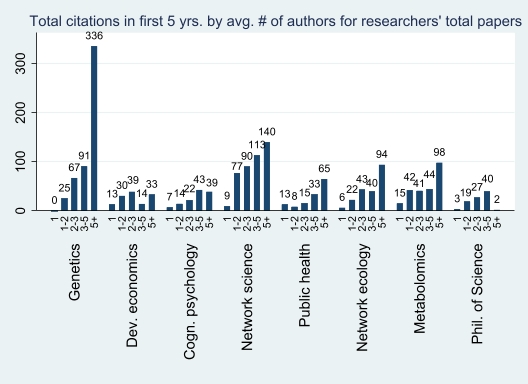
**

**Supplementary Figure S3. Total citations in first 5 years by average number of authors for researchers’ total papers in first 5 years, for the 100 prominent researchers across fields.**

**
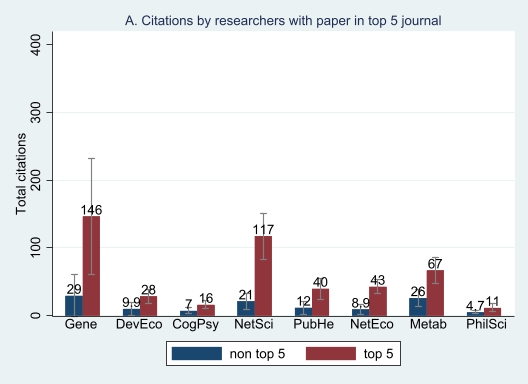

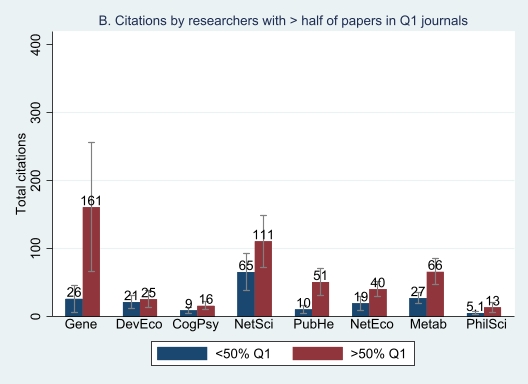
**


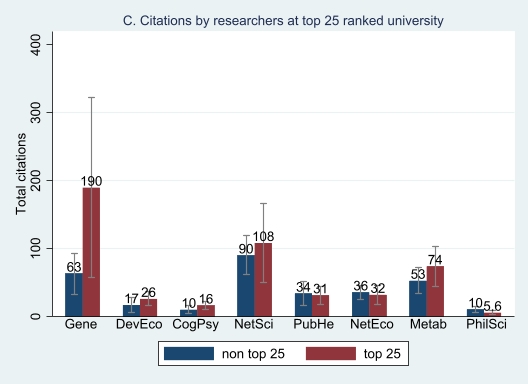

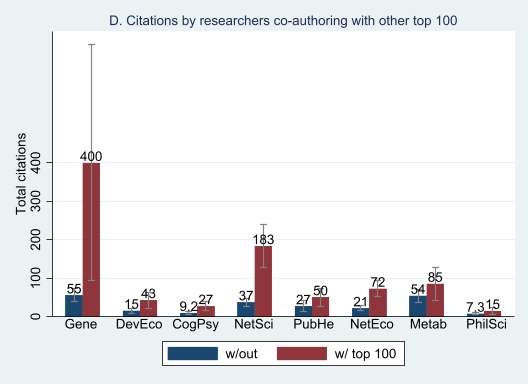


**Supplementary Figure S4. Early-career factors of total citations in the first 5 years for the 100 prominent researchers across fields.** All data reflect the first 5 years of researchers’ careers since their first publication. >50% in Q1 refers to all researchers who have more than half of all their papers published in first quartile journals, and <50% in Q1 refers to all (other remaining) researchers who have less than half of all their papers published in first quartile journals. Confidence intervals are reported at the 95% level. Researchers with any of these four key features have consistently accrued many more citations in the first five years of their career across all fields, providing evidence for our jump start hypothesis. Researchers with a paper published in a top 5 journal within the first five years received at least 4 times more citations than those without such a publication in fields like genetics and network science. Co-authorship with prominent researchers has particularly high returns on citations within fields like genetics and network science.


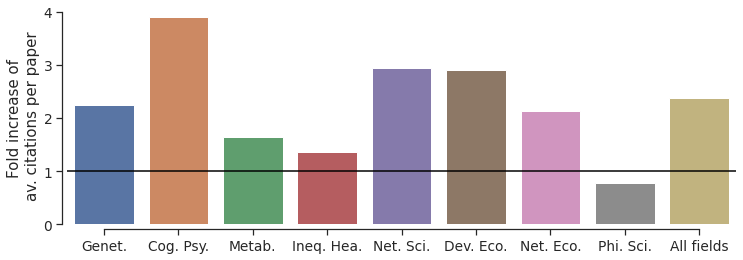


**Supplementary Figure S5. Fold increase of average citations per paper in collaboration with other prominent scientists during the first 5 years of researchers’ career.** For each field, and for all fields combined, each bar shows the ratio between the number of citations per paper, in which a researcher collaborates with other prominent scientists in their field and the citations per paper for those papers not in collaboration with other prominent scientists. The black line indicates no fold increase. Bars above 1 show increased citations in papers with prominent collaboration, whereas bars below 1 indicate no increase. Note that in all fields except for philosophy of science, there is an increase in the number of average citations per paper. Overall, papers published with other prominent scientists during the first 5 years of their career receive over two times more citations than those papers not in collaboration over that period of time.

**
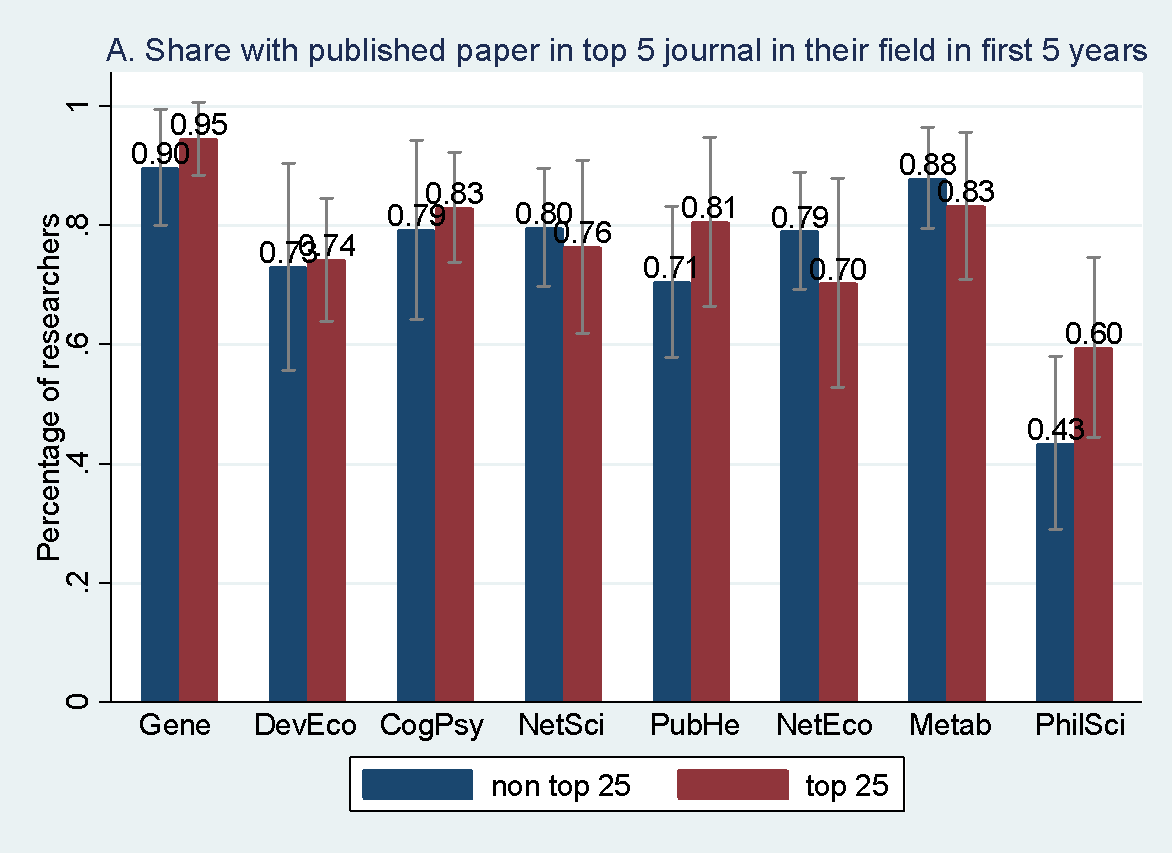

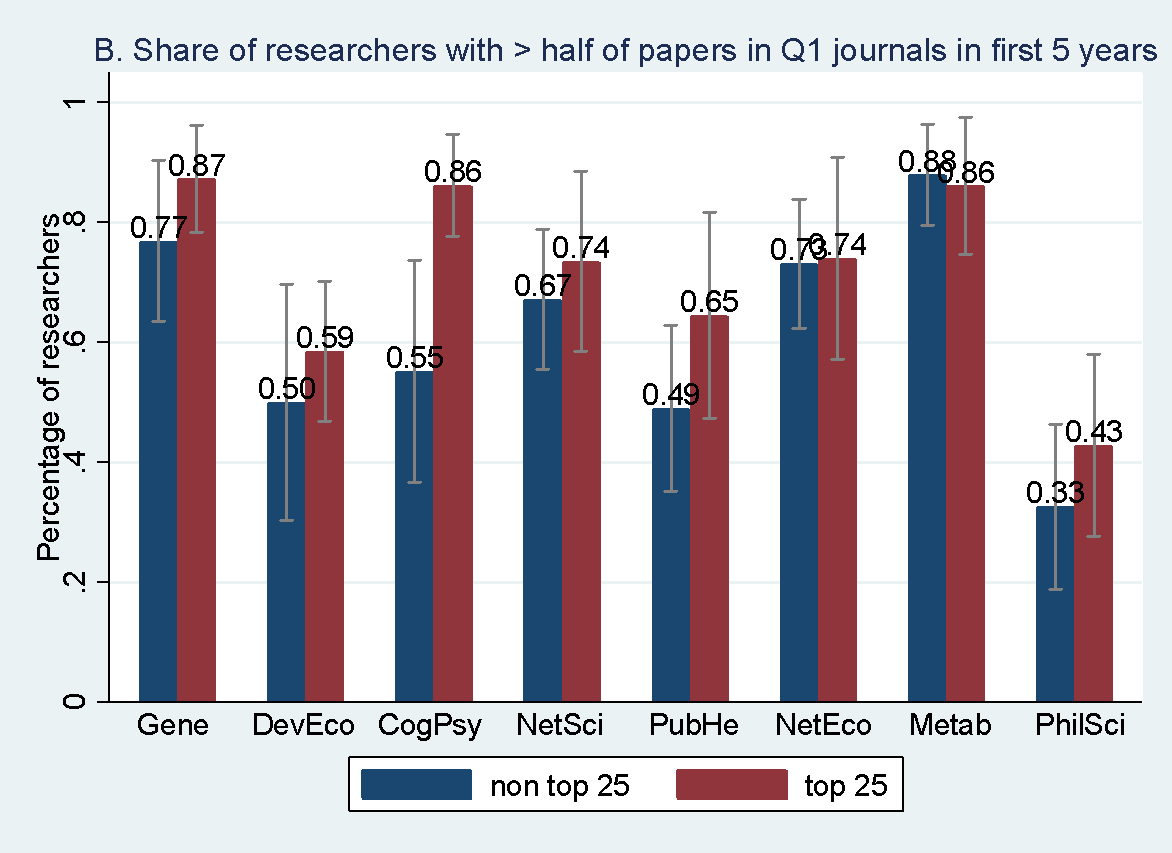

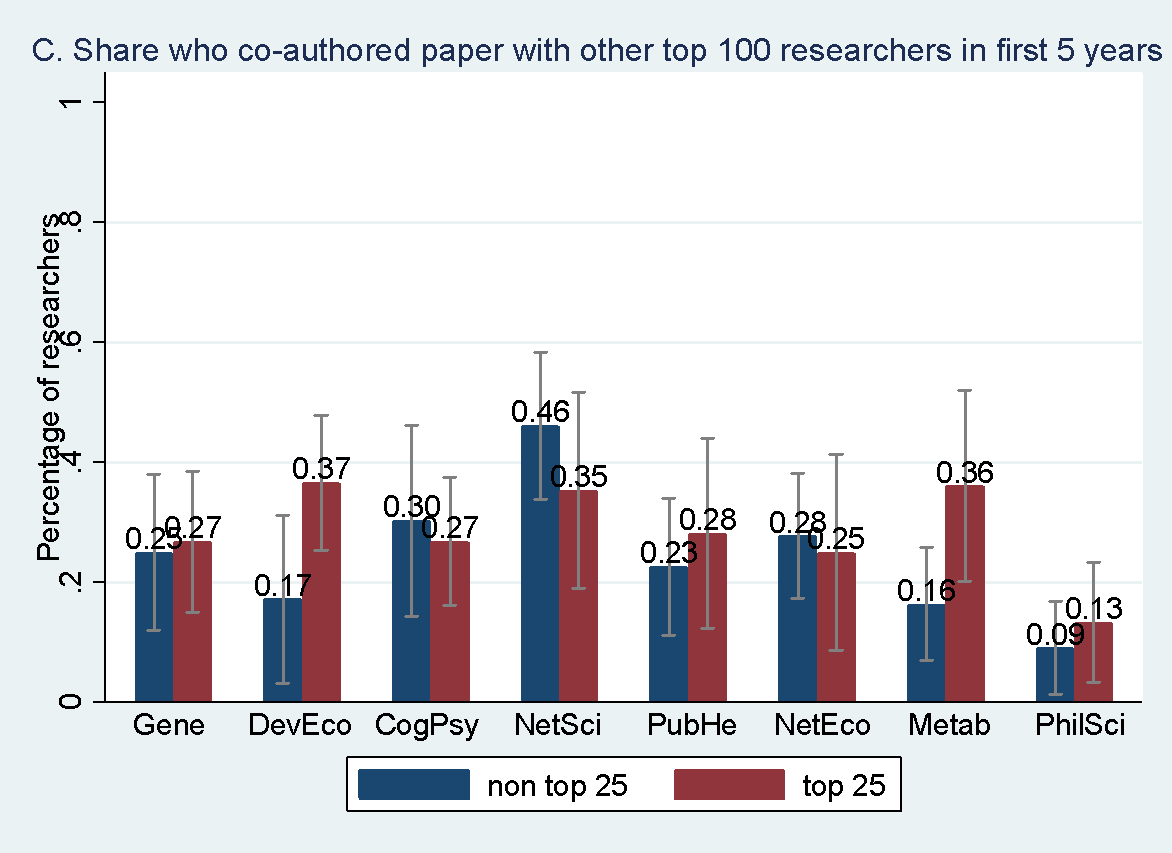
**

**Supplementary Figure S6. Affiliation at a top 25 university in the first 5 years among the 100 prominent researchers by their publication and collaboration patterns, across fields.** Confidence intervals are reported at the 95% level.

**
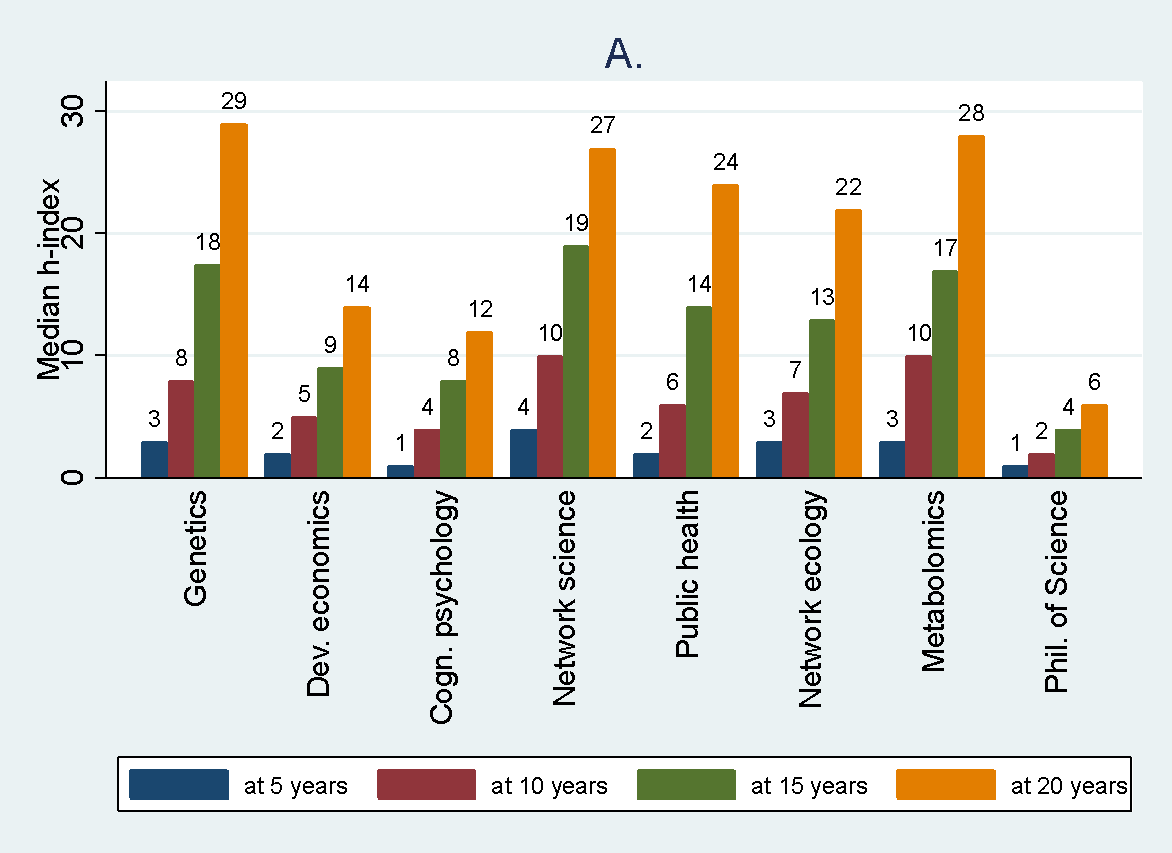

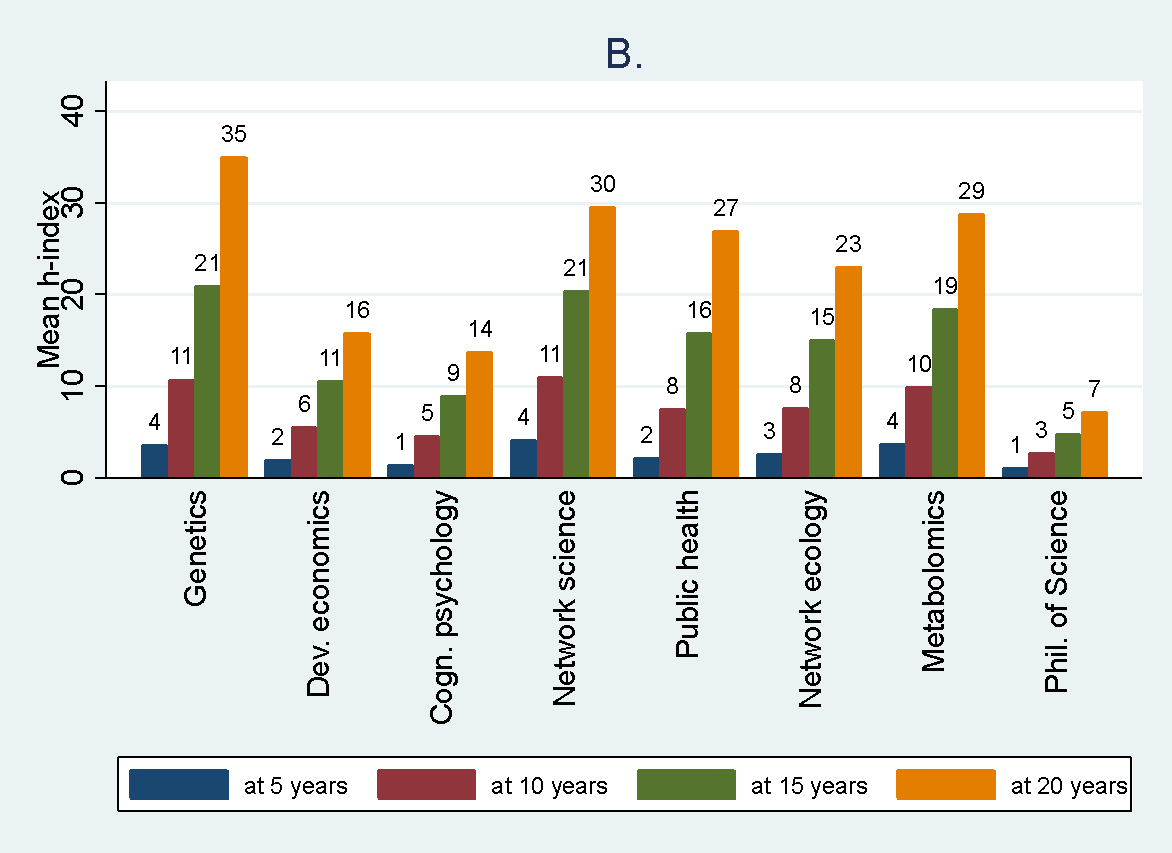
**

**Supplementary Figure S7. Changes in the 100 prominent researchers’ h-index at 5, 10, 15 and 20 years since their first publication, across 8 fields.** A. Median h-index. B. Mean h-index.


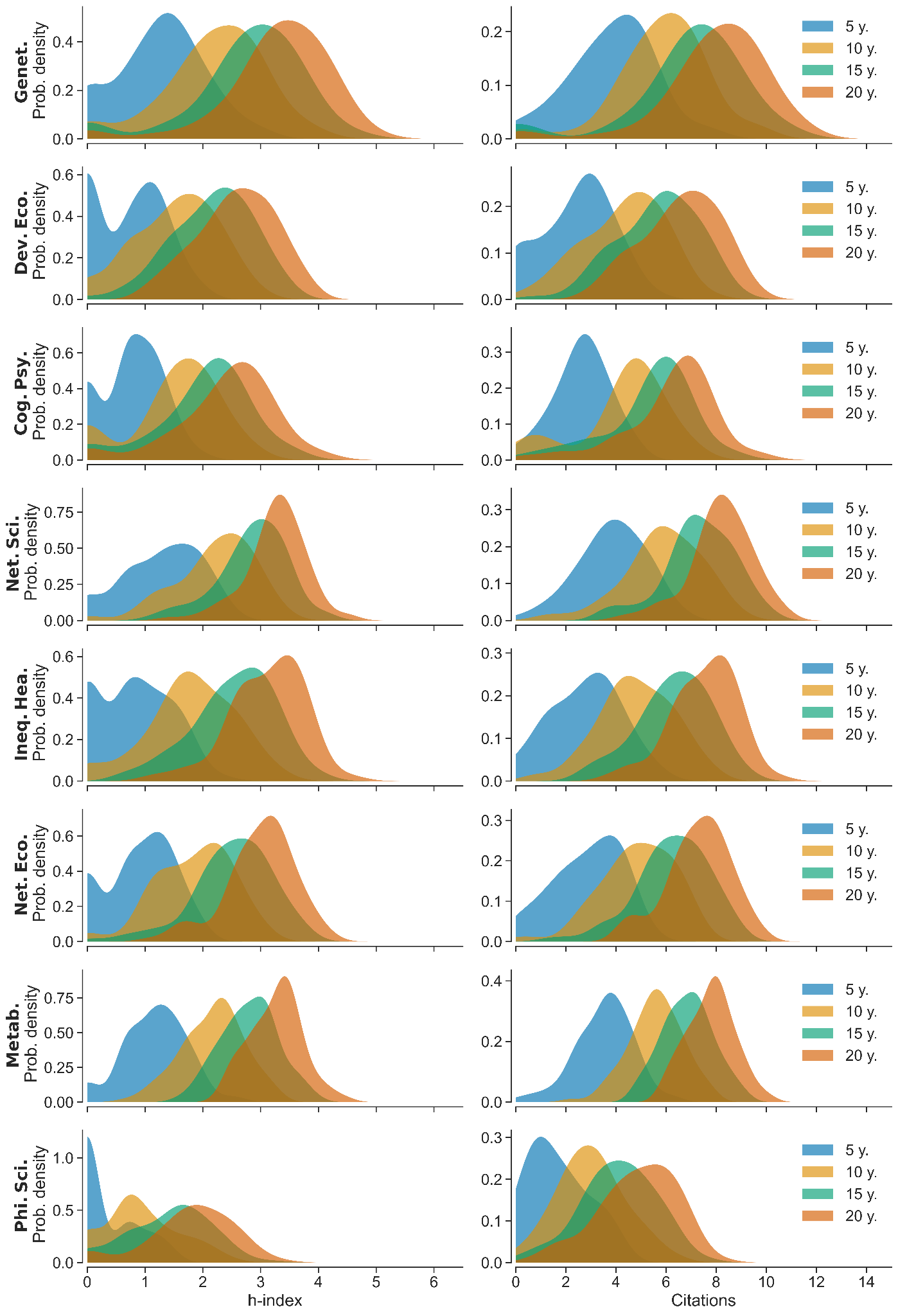


**Supplementary Figure S8. Distribution of h-index and received citations across fields and researchers’ career.** H-index and number of citations distribution (logarithmic scale) at 5, 10, 15 and 20 years since the first publication.


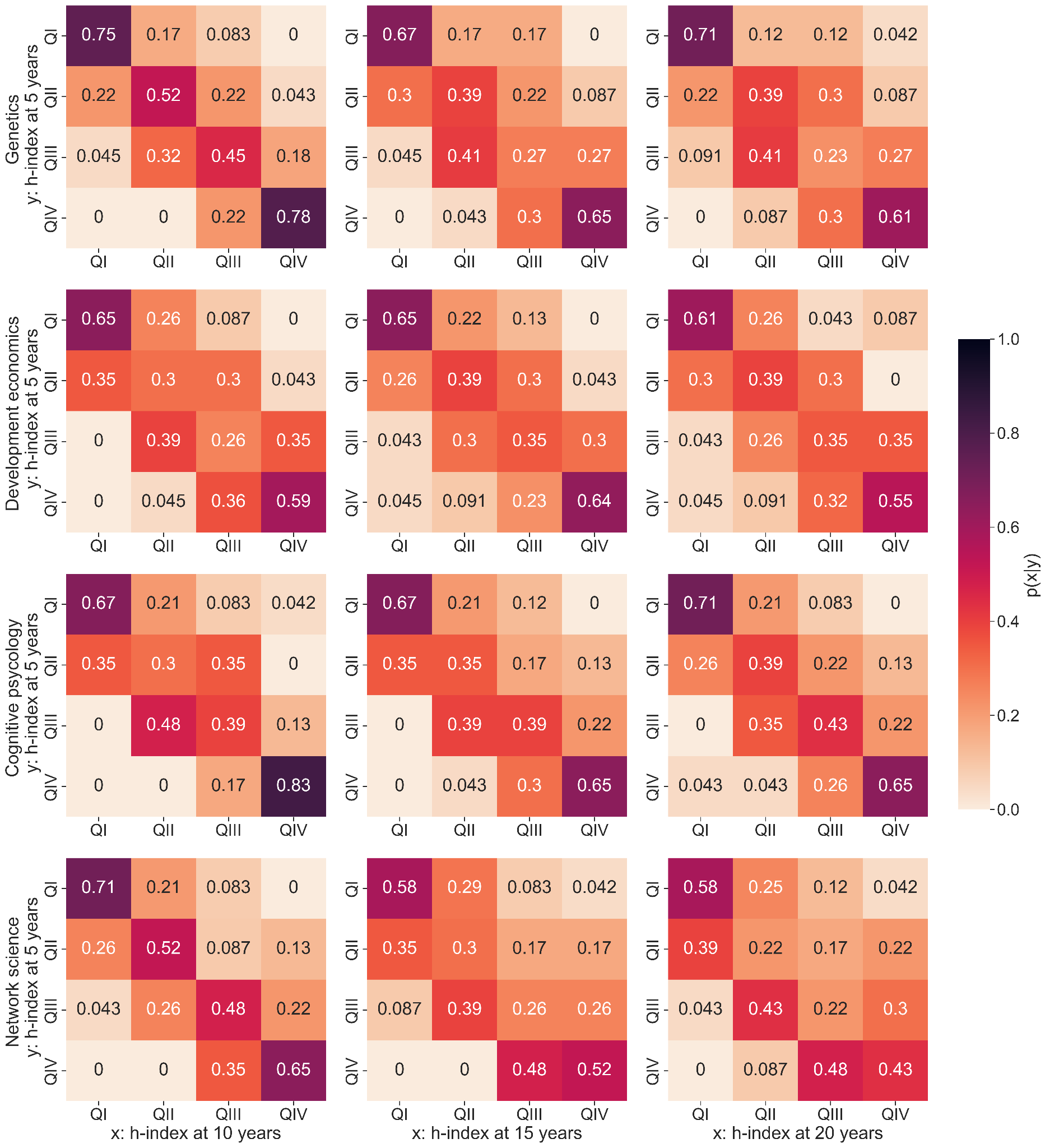

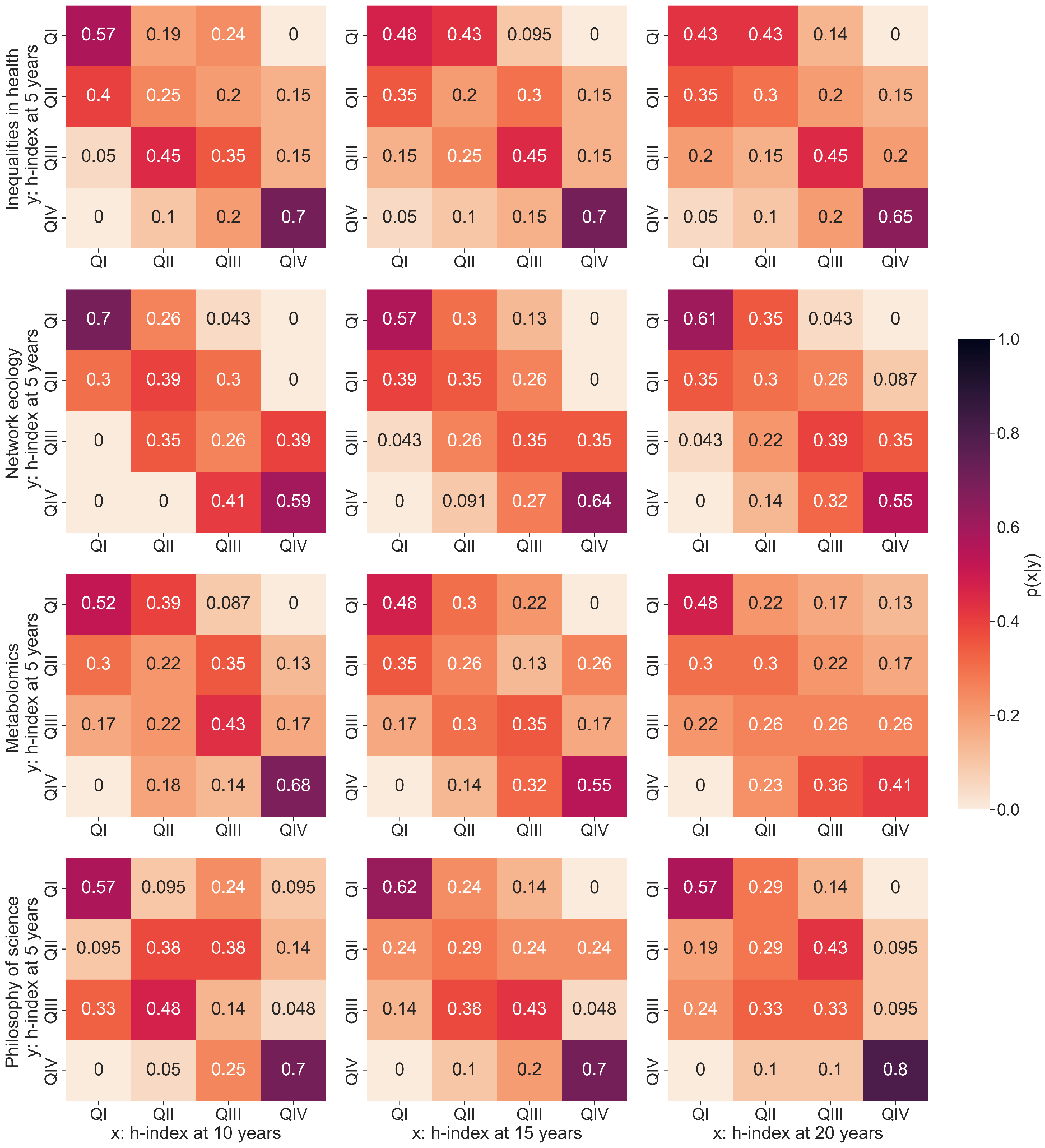


**Supplementary Figure S9. Evolution of h-index quartiles across researchers’ career.** For each field, we show the transition matrices from h-index quartile at five years to h-index quartiles at 10, 15 and 20 years since the first publication. Each element (i,j) of a transition matrix shows the probability that a researcher who was in quartile Qi at 5 years is in quartile Qj at 10, 15 and 20 years since the first publication. Each matrix element is colour coded according to the colour bar on the right.


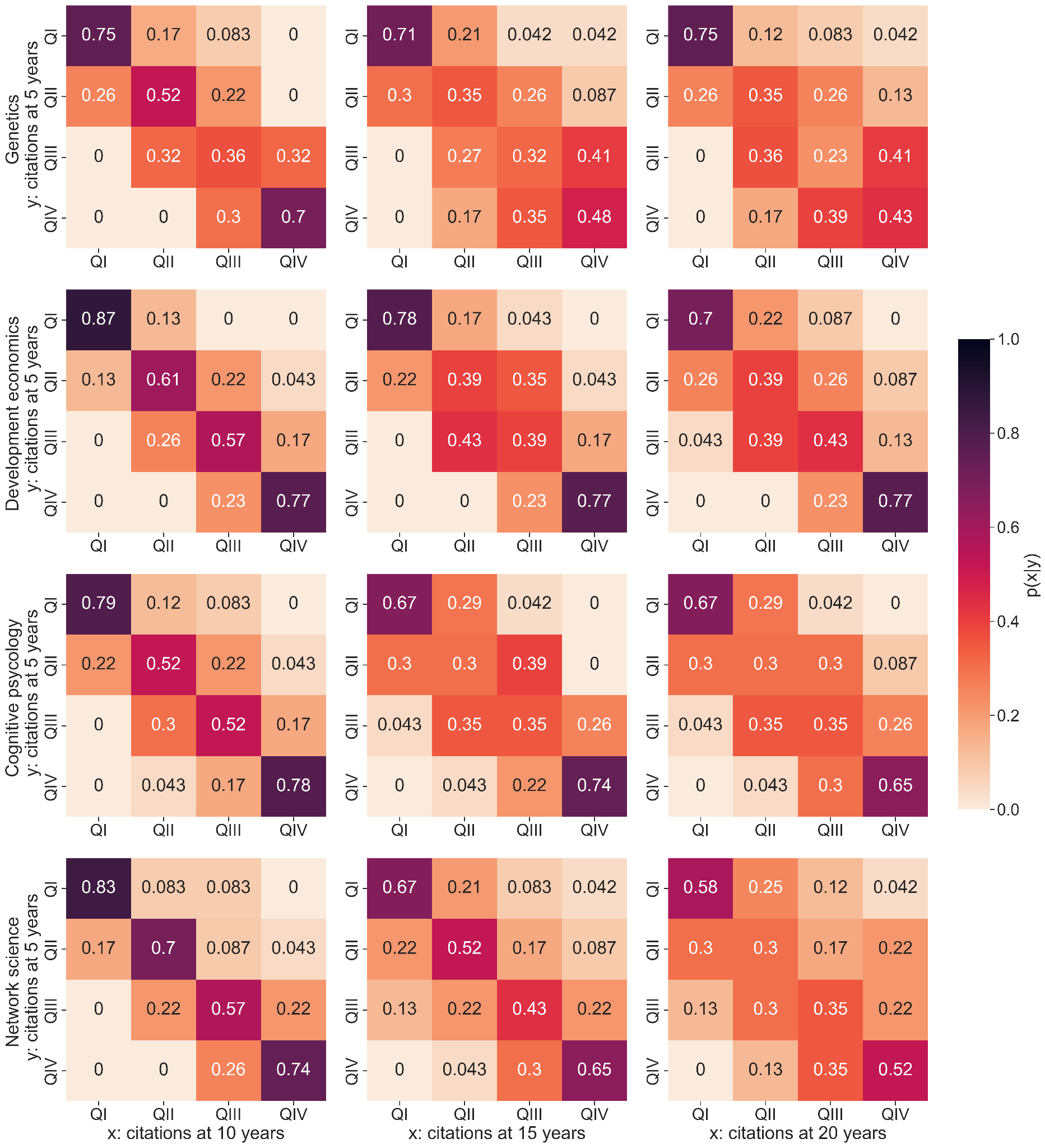

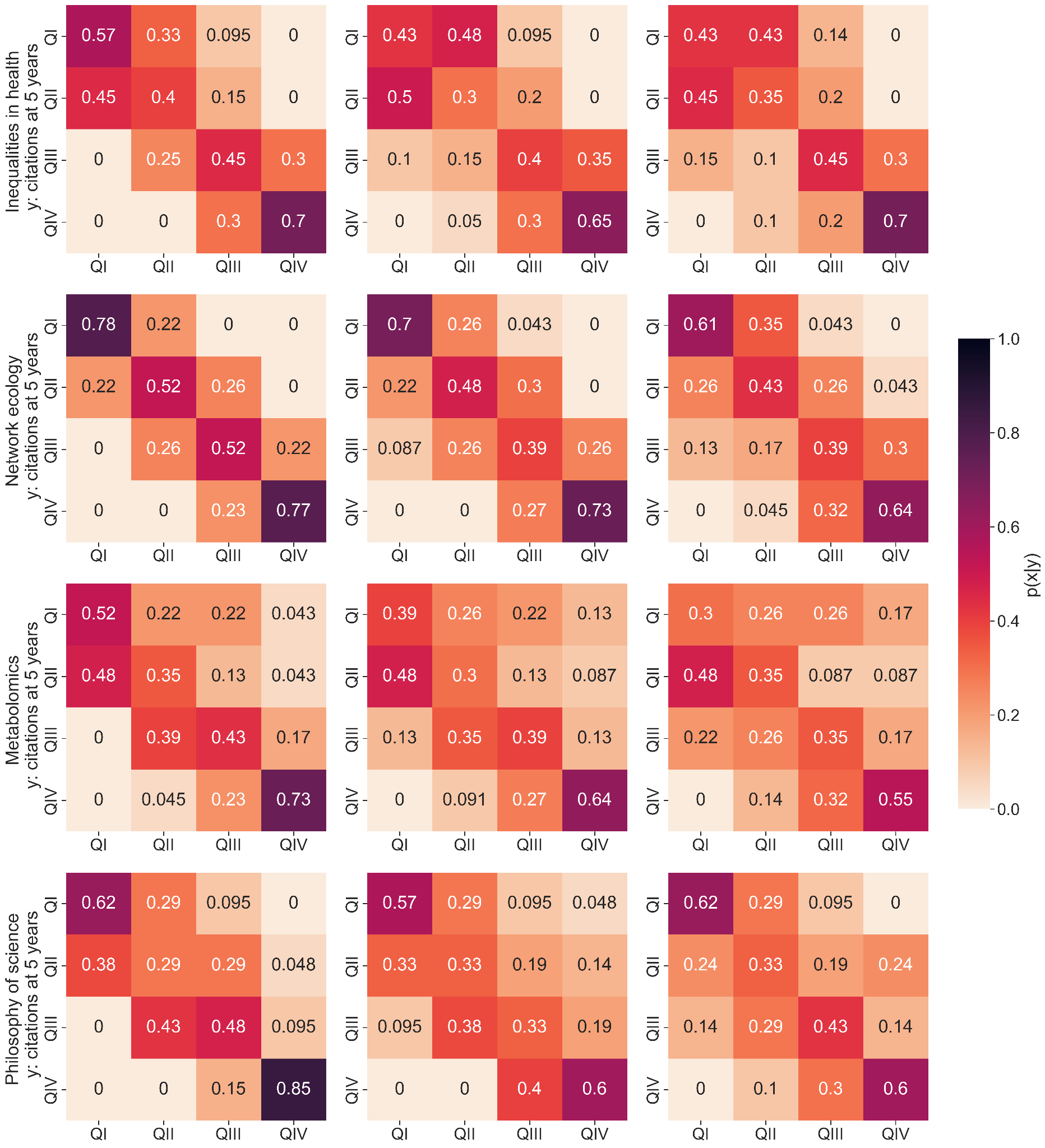


**Supplementary Figure S10. Evolution of citation quartiles across researchers’ career.**  We show the transition matrices from h-index quartile at five years to h-index quartiles at 10, 15 and 20 years since the first publication. Each element (i,j) of a transition matrix shows the probability that a researcher who was in quartile Qi at 5 years is in quartile Qj at 10, 15 and 20 years after the first publication. Each matrix element is colour coded according to the colour bar on the right.


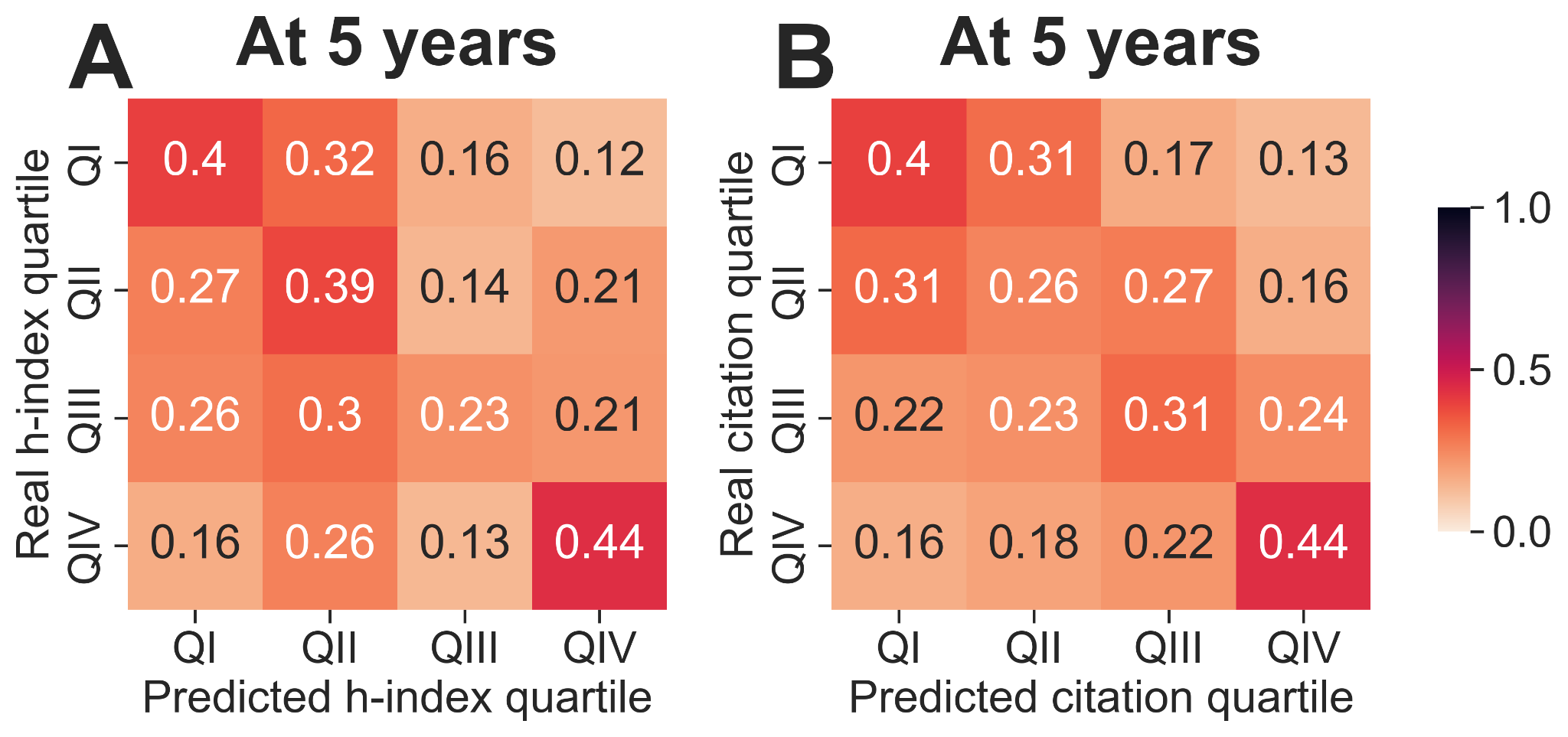


**Supplementary Figure S11. Confusion matrices for predicting h-index and citation quartile with model 2.** (**A**) H-index quartile at 5 years, and (**B**) citation quartile at 5 years. These matrices show, for each row Qi, the fraction of researchers in Qi at 5 years who are classified as QI, QII, QII and QIV.


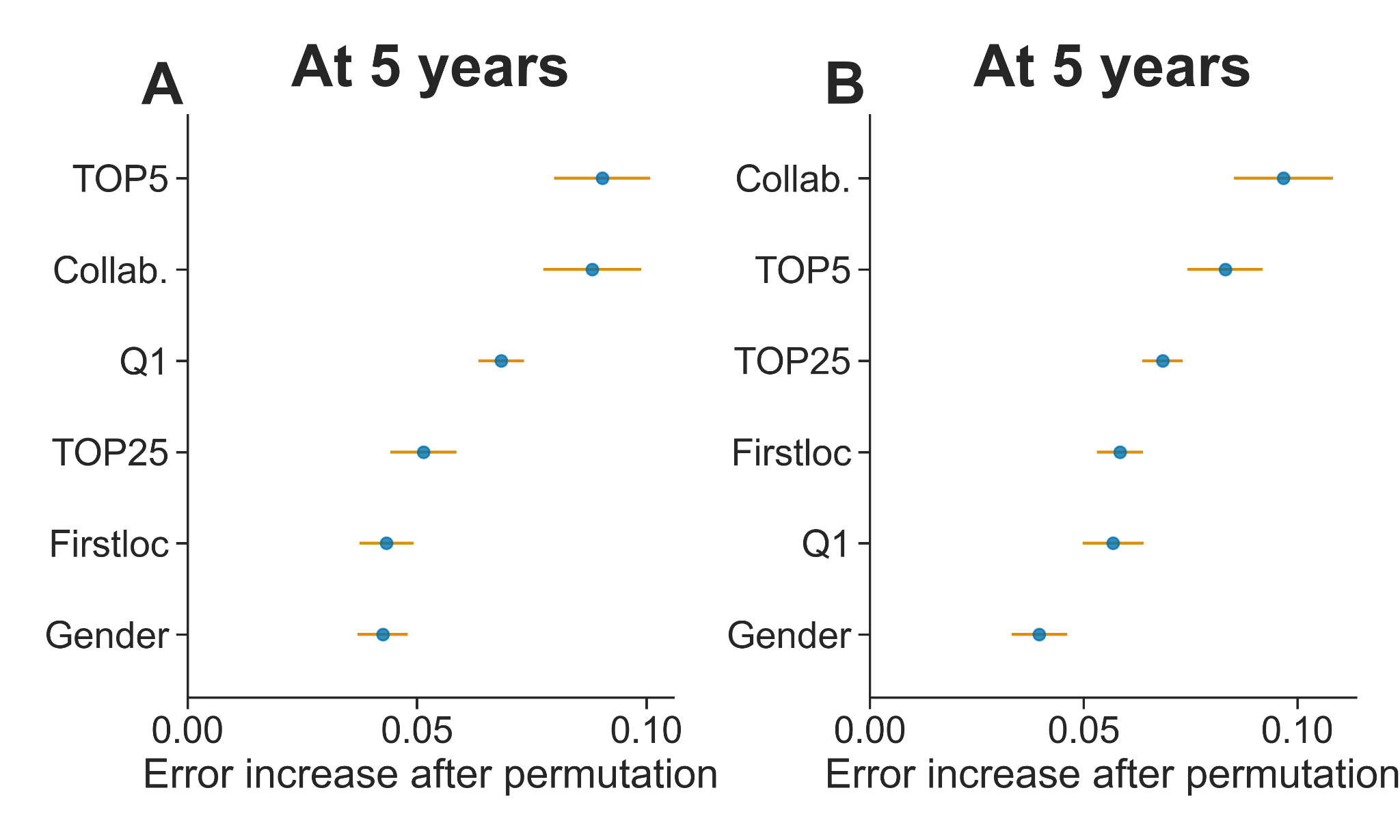


**Supplementary Figure S12. Parameter importance for predicting quartiles at 5 years.** Importance for h-index (**A**) and citation (**B**) quartile prediction. Parameter importance is an estimate of the increase in prediction error when we remove that feature from the classifier. The greater the error increase, the greater the importance of the parameter.


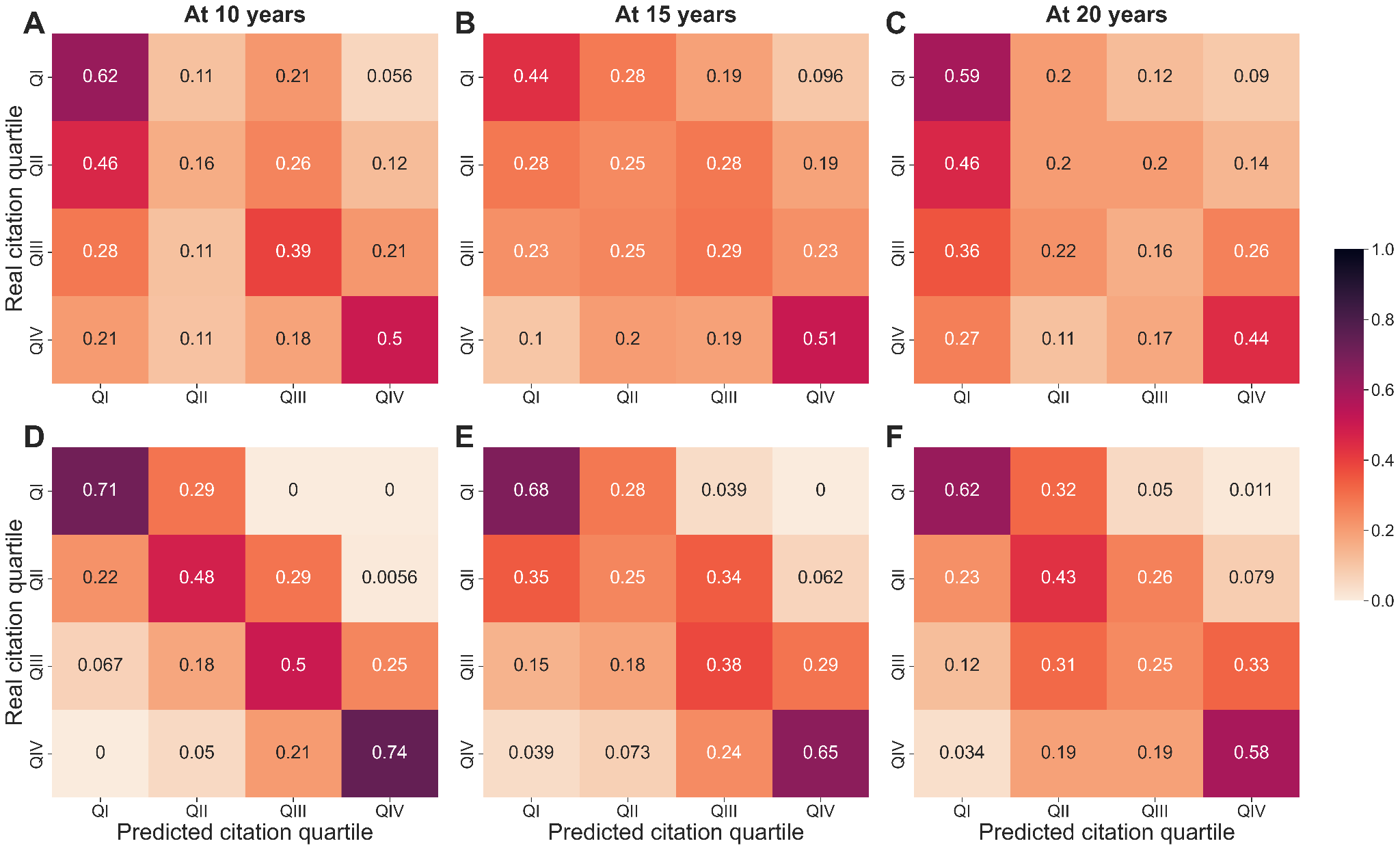


**Supplementary Figure S13. Confusion matrices for predicting citation quartiles with models 2 and Q5.** Results reflect the same analysis as in Fig. 5 of the main text but for the prediction of citation quartile. The first row (**A-C**) corresponds to model 2 and the second row (**D-F**) to model Q5. These matrices show, for each row Qi, the fraction of researchers in Qi at 10, 15 or 20 years that are classified as QI, QII, QII and QIV by each model. The darker the region along the diagonal, the higher the coincidence between the classifier and the real data.


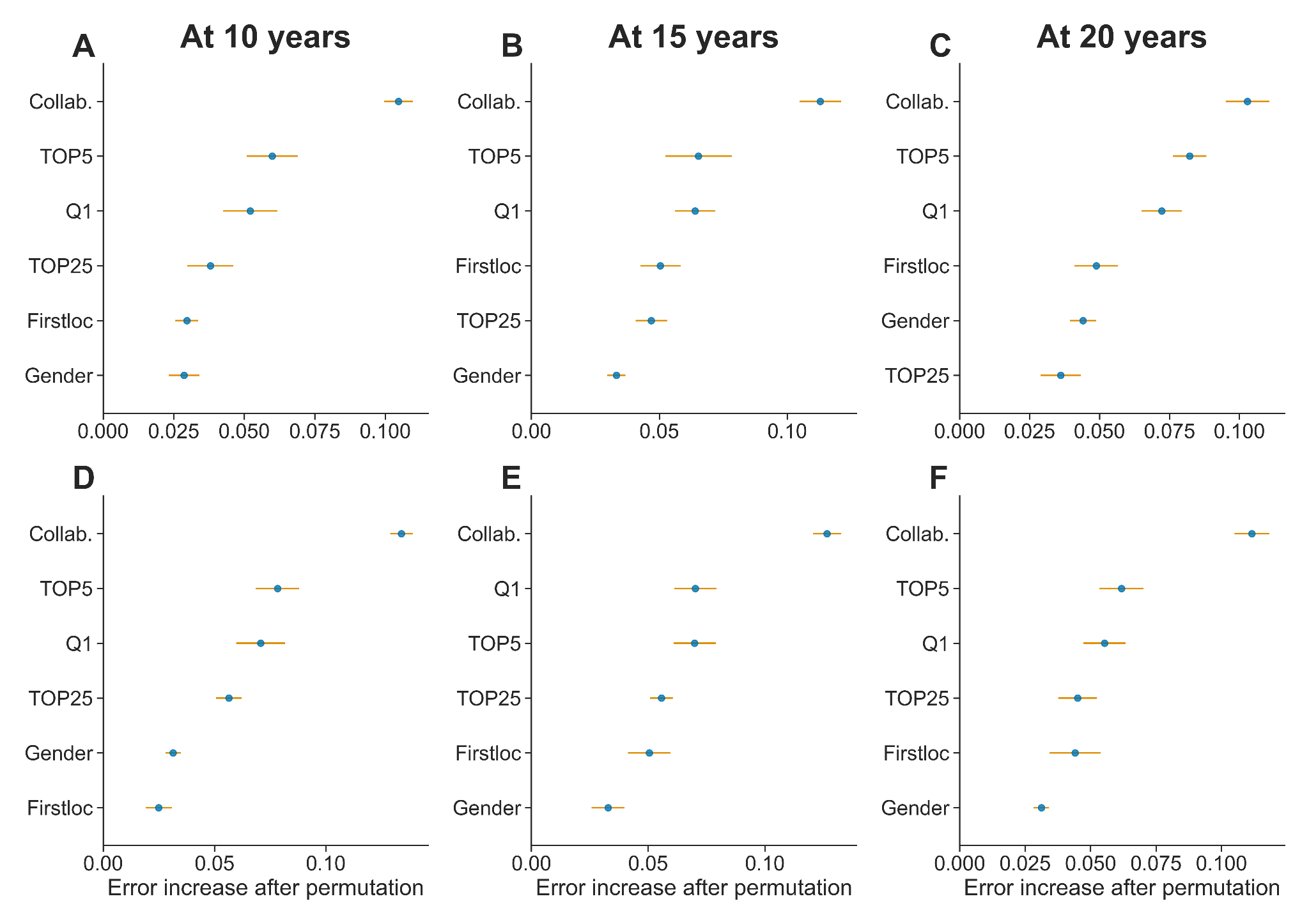


**Supplementary Figure S14. Parameter importance for predicting h-index and citation quartile at 10, 15 and 20 years with model 2.** The first row (**A-C**) corresponds to parameter importance for h-index quartile prediction and the second row (**D-F**) for predicting citation quartile.


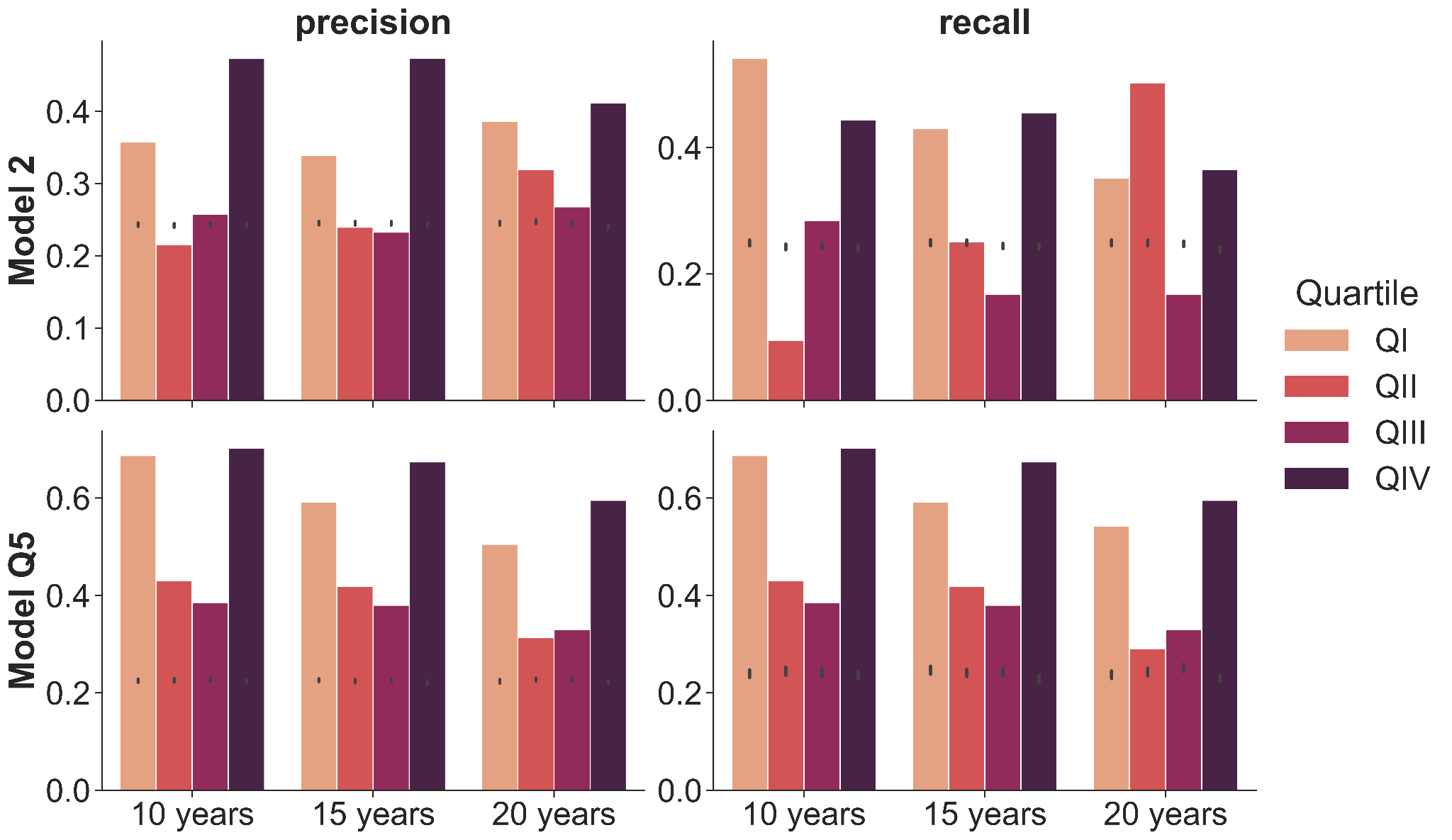


**Supplementary Figure S15. Precision and recall for predicting h-index quartiles.**  We show the precision and recall metrics when predicting h-index quartile at 10, 15 and 20 years. Black points represent the 95% confidence interval expected when predicting randomly assigned h-index quartiles.


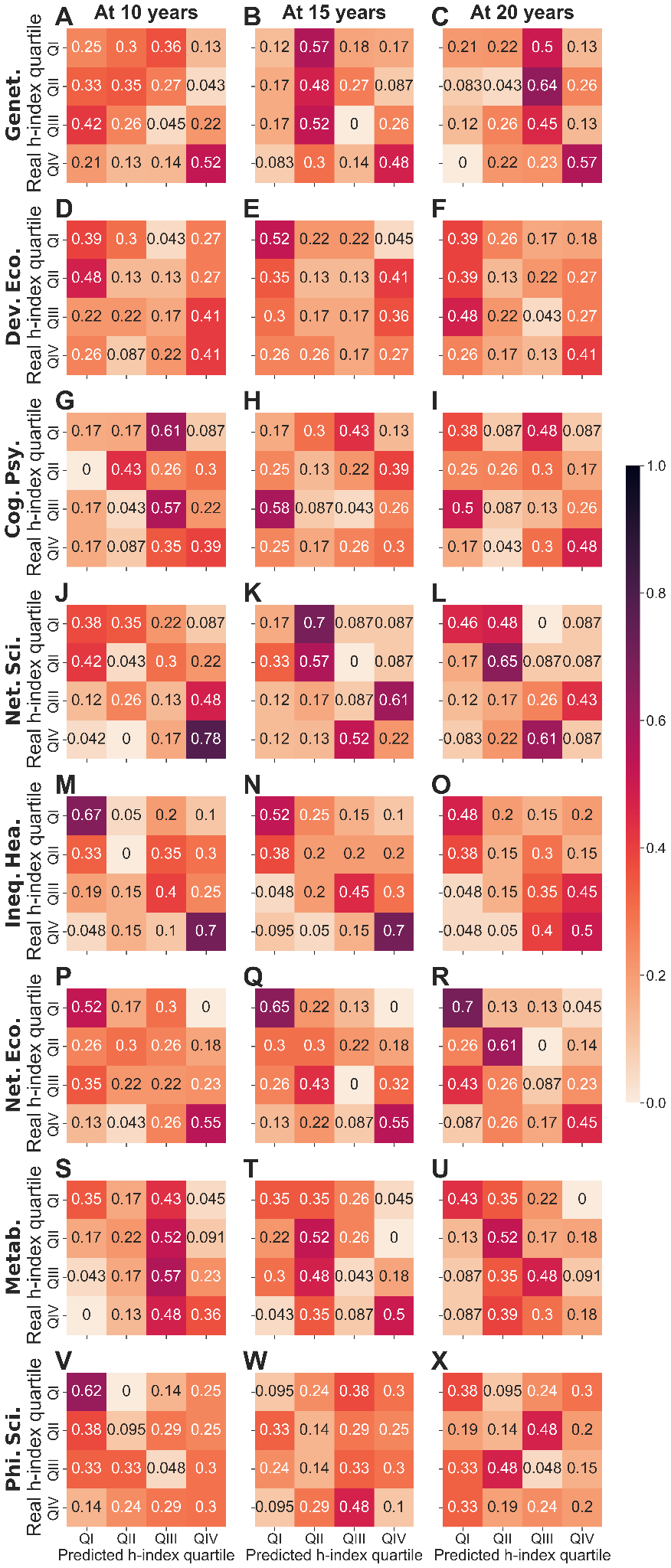


**Supplementary Figure S16. Confusion matrices of h-index per field for model 1.** Confusion matrices for the output of the classifier for model 1. These matrices show, for each row Qi, the fraction of researchers in Qi at 10, 15 or 20 years that are classified as QI, QII, QII and QIV by each model. The darker the region, the higher the coincidence between the classifier and the real data.


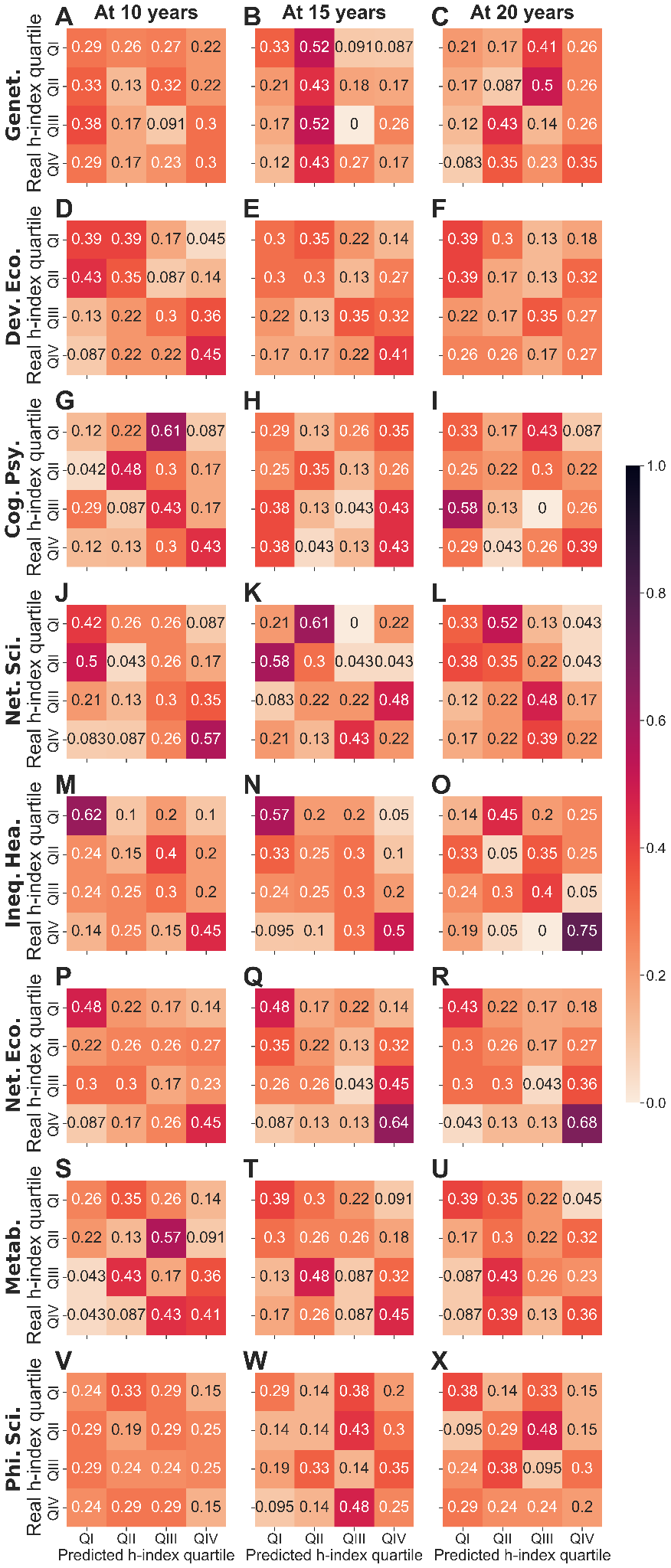


**Supplementary Figure S17. Confusion matrices of h-index per field for model 2.** Confusion matrices for the output of the classifier for model 2. The darker the region, the higher the coincidence between the classifier and the real data.


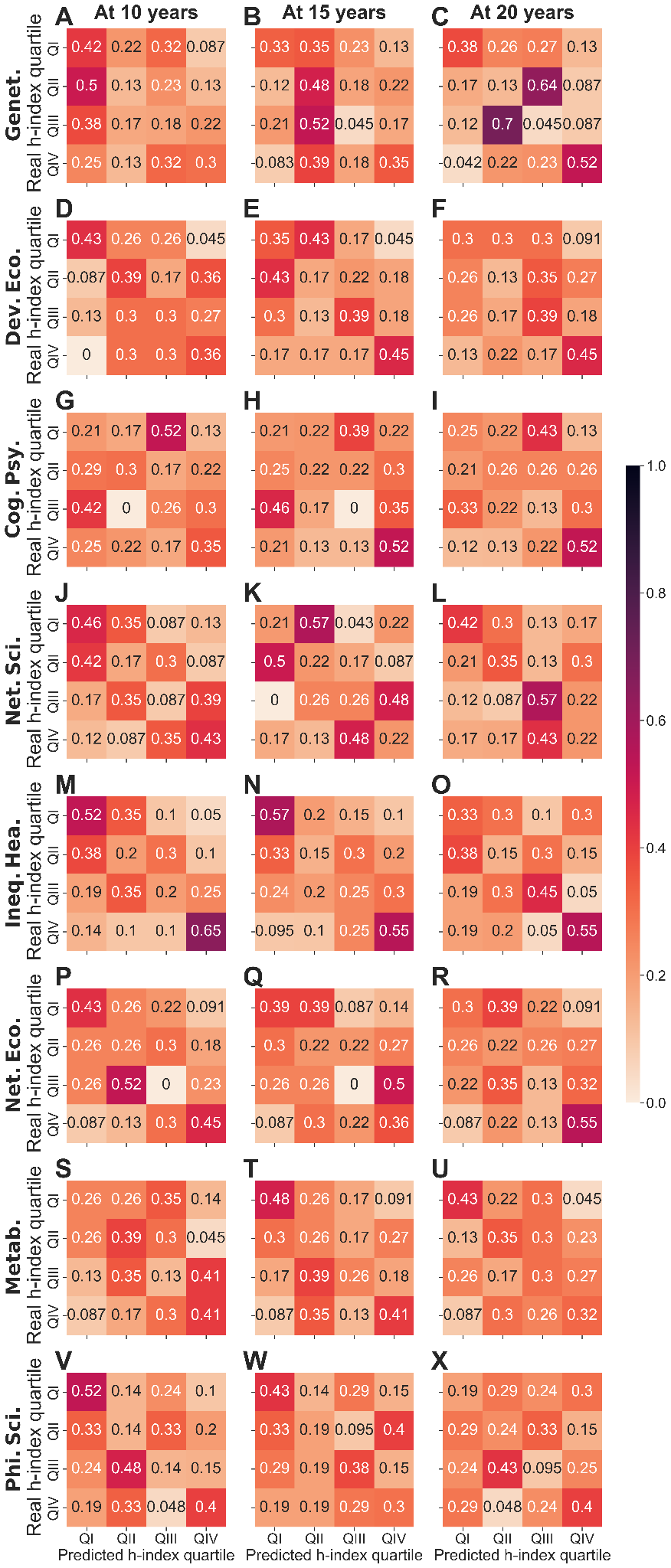


**Supplementary Figure 18. Confusion matrices of h-index per field for model 3.** Confusion matrices for the output of the classifier for model 3. The darker the region, the higher the coincidence between the classifier and the real data.


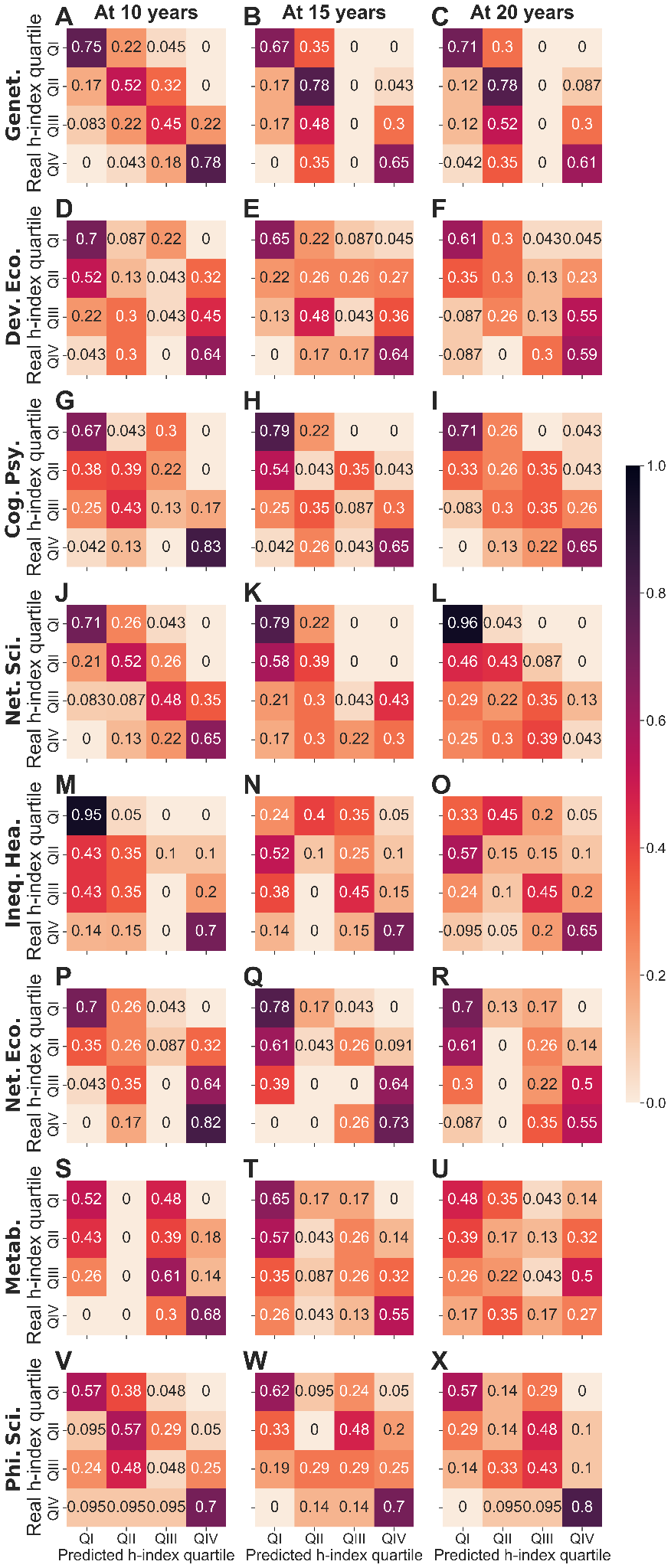


**Supplementary Figure S19. Confusion matrices of h-index per field for model Q5.** Confusion matrices for the output of the classifier for model Q5. The darker the region, the higher the coincidence between the classifier and the real data.


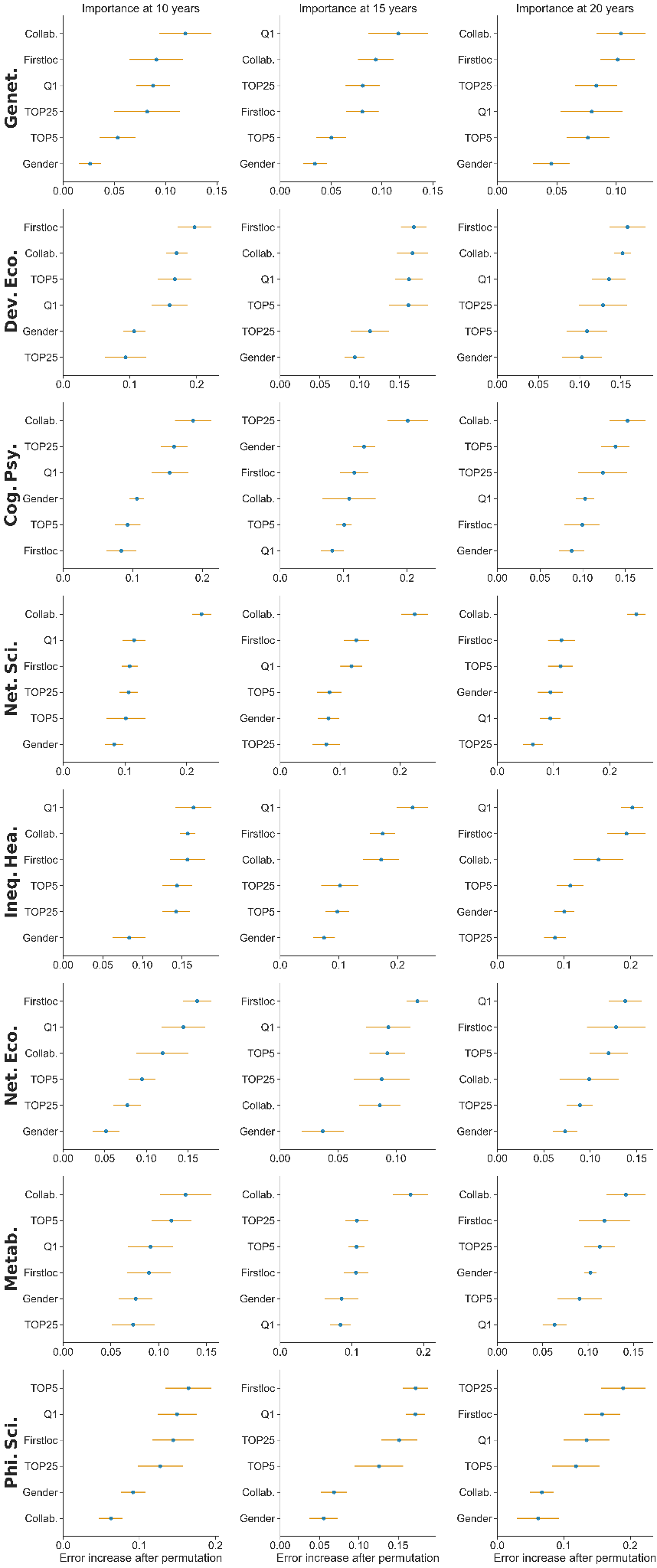


**Supplementary Figure S20. Parameter importance for predicting h-index quartile.** Importance obtained from the RF classifier for predicting h-index quartile at 10, 15 and 20 years.


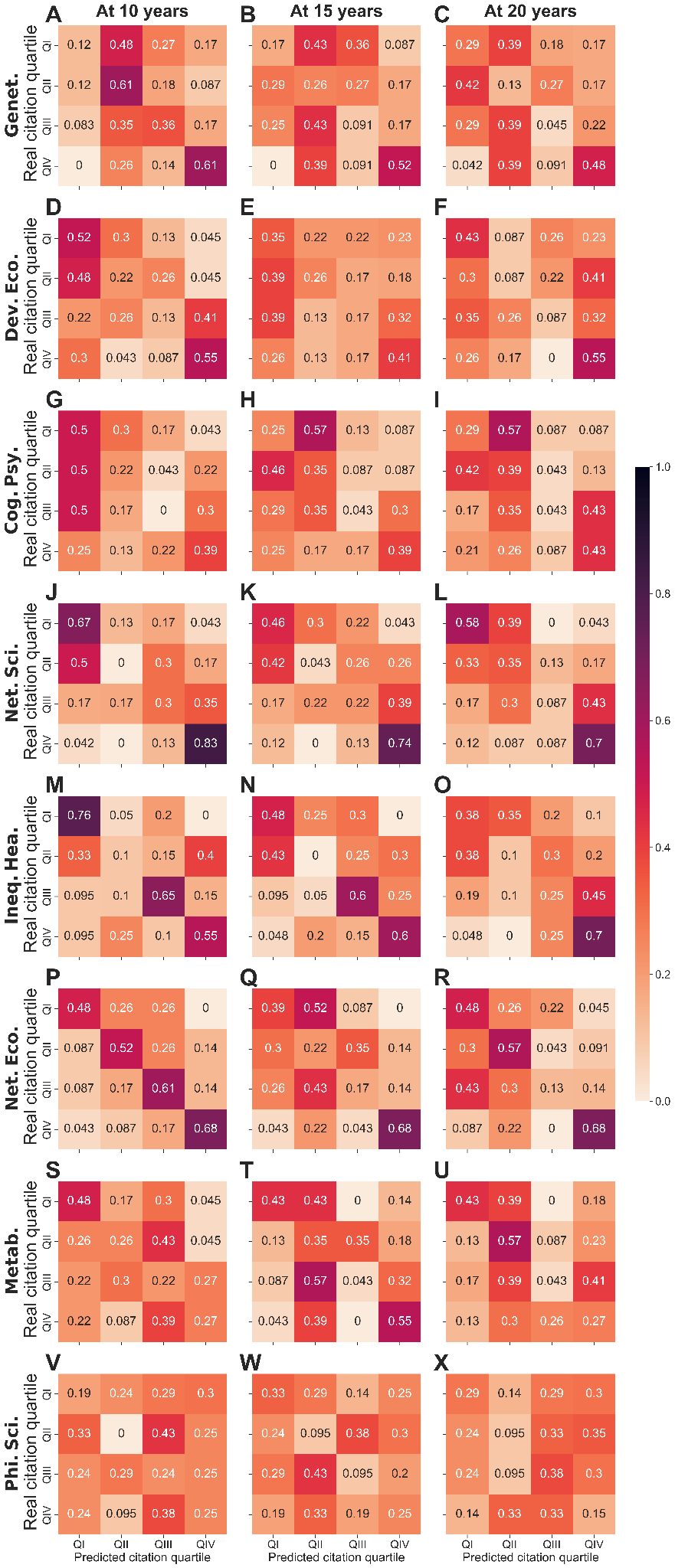


**Supplementary Figure S21. Confusion matrices of citations per field for model 1.** Confusion matrices for the output of the classifier for model 1 predicting citation quartiles at 10, 15 and 20 years. The darker the region, the higher the coincidence between the classifier and the real data.


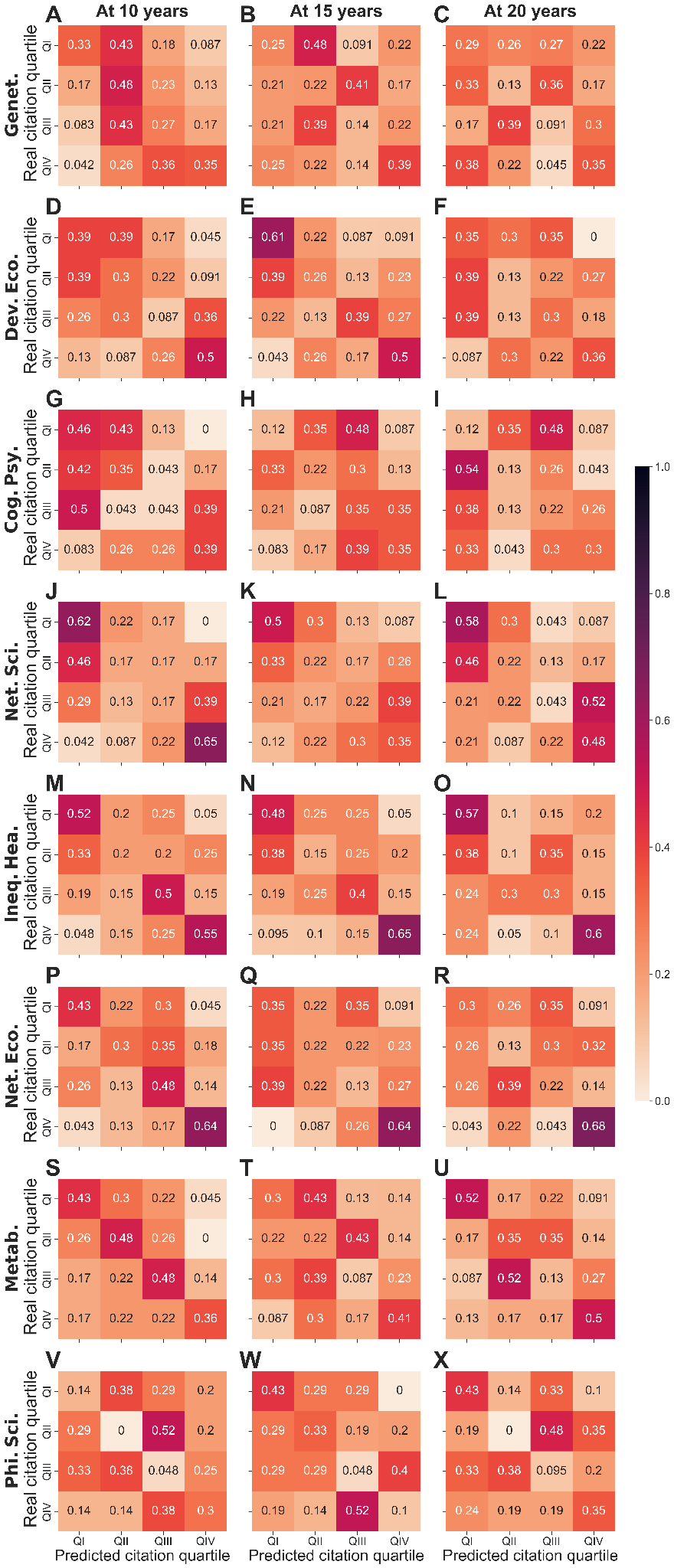


**Supplementary Figure S22. Confusion matrices of citations per field for model 2.** Confusion matrices for the output of the classifier for model 2 predicting citation quartile at 10, 15 and 20 years. The darker the region, the higher the coincidence between the classifier and the real data.


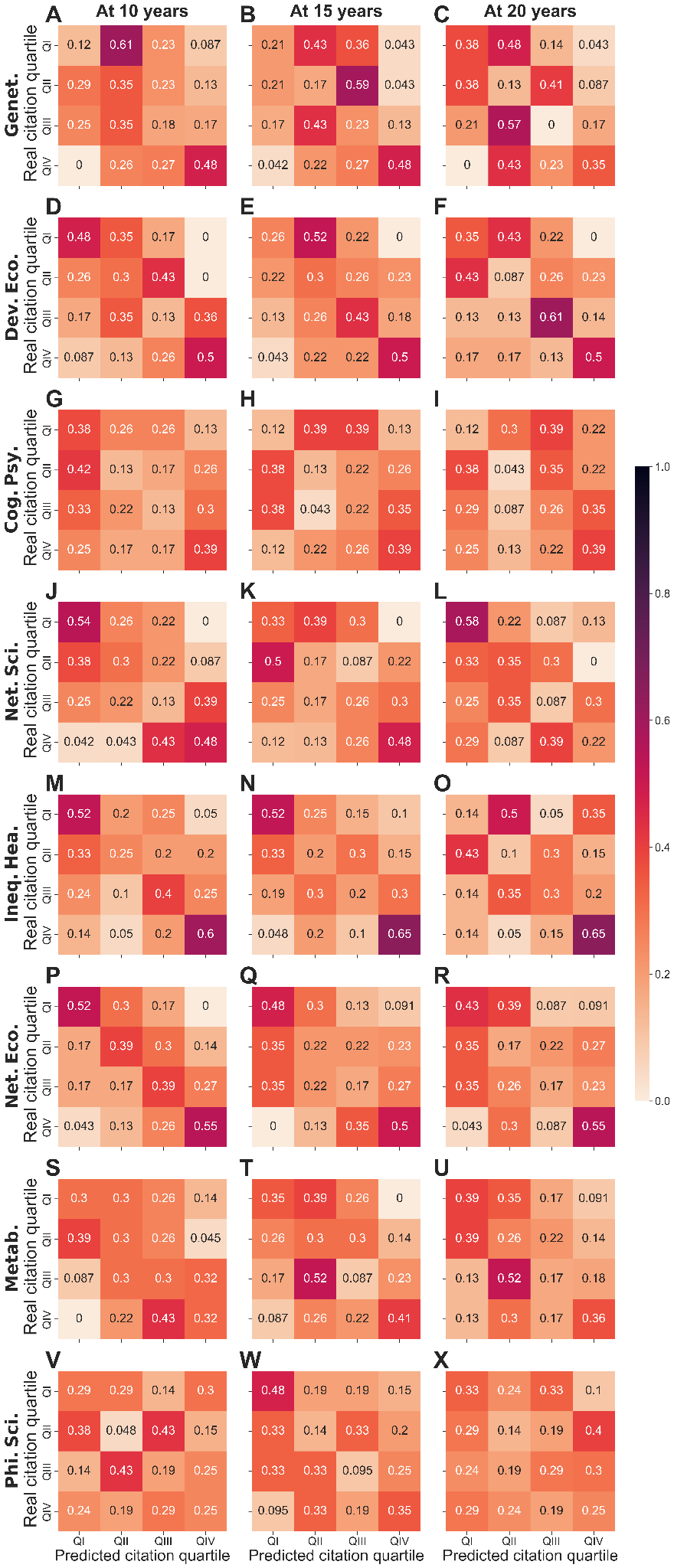


**Supplementary Figure S23. Confusion matrices of citations per field for model 3.** Confusion matrices for the output of the classifier for model 3 predicting citation quartile at 10, 15 and 20 years. The darker the region, the higher the coincidence between the classifier and the real data.


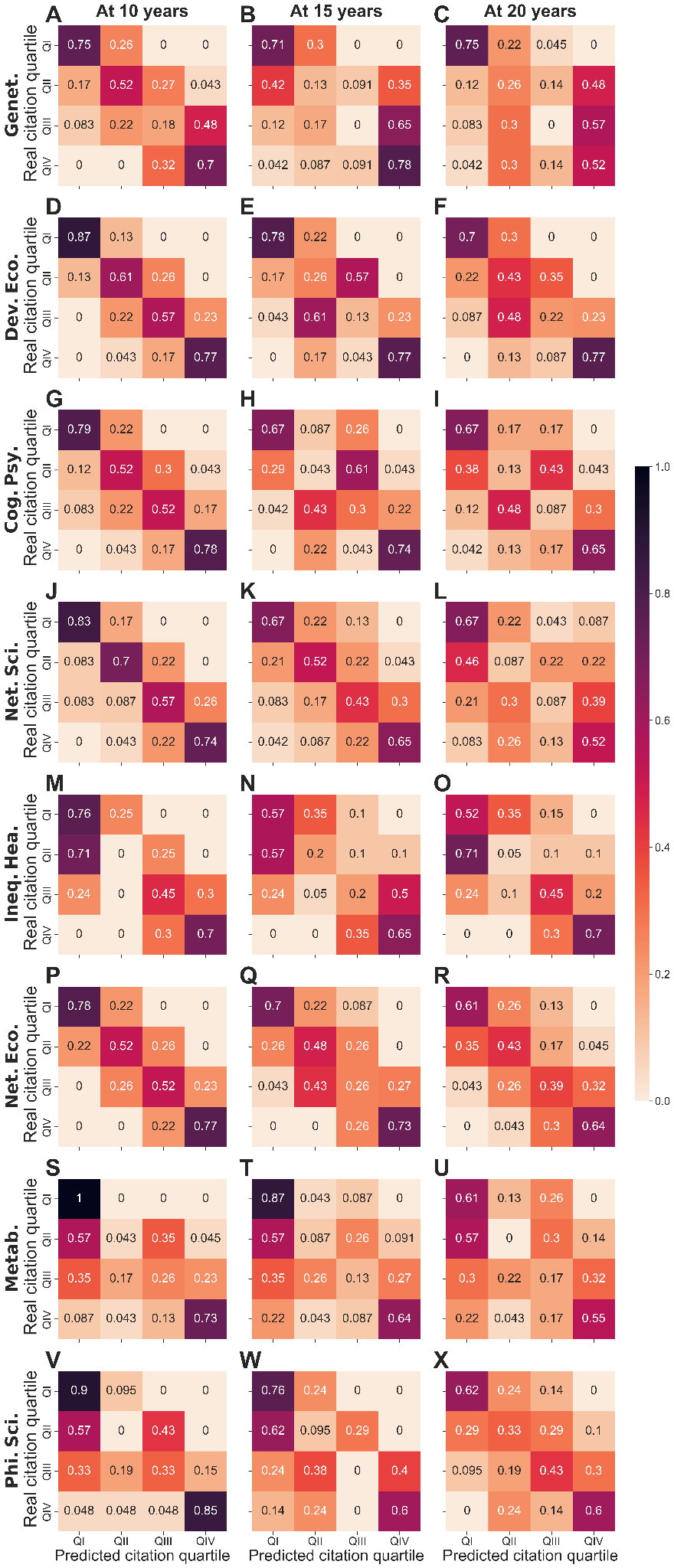


**Supplementary Figure S24. Confusion matrices of citations per field for model Q5.** Confusion matrices for the output of the classifier for model Q5 predicting citation quartile at 10, 15 and 20 years. The darker the region, the higher the coincidence between the classifier and the real data.


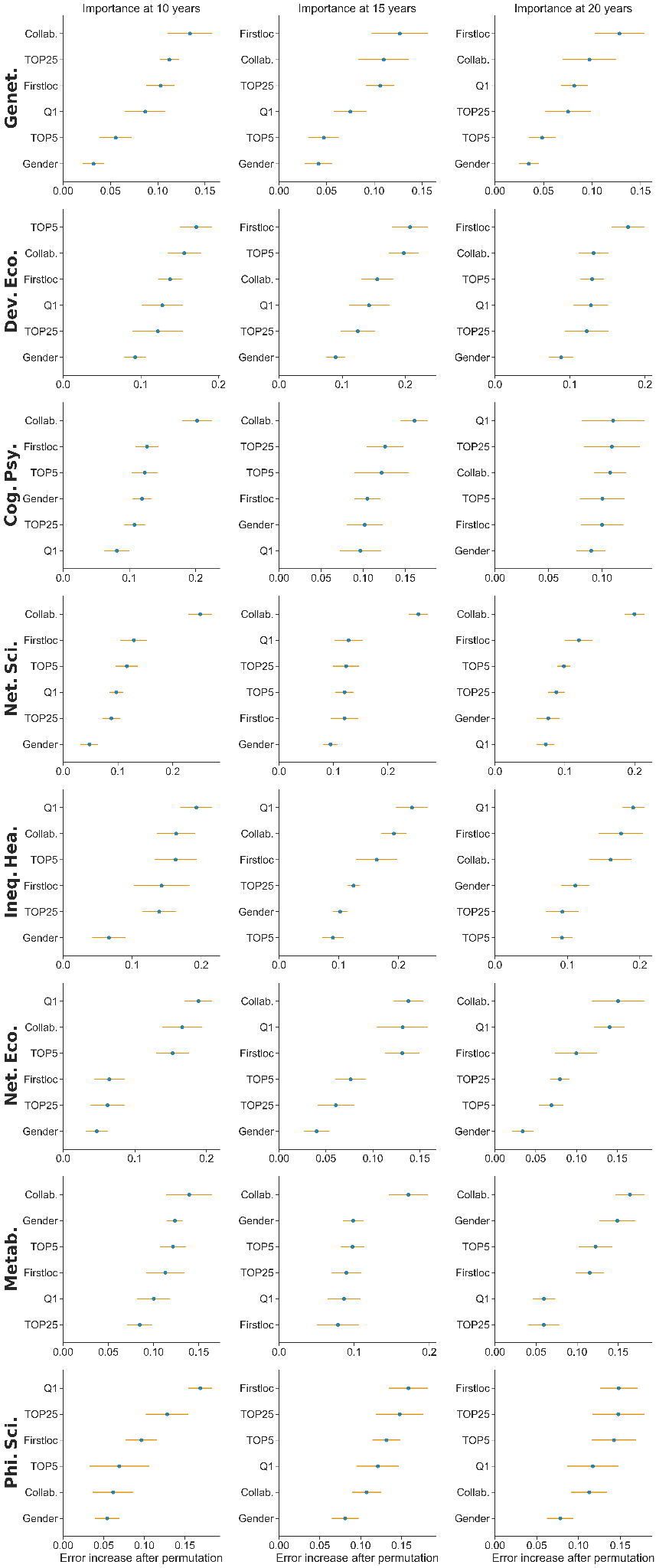


**Supplementary Figure S25. Parameter importance for predicting citation quartile.** Importance obtained from the RF classifier for predicting h-index quartile at 10, 15 and 20 years. The greater the error increase, the more important the parameter is.


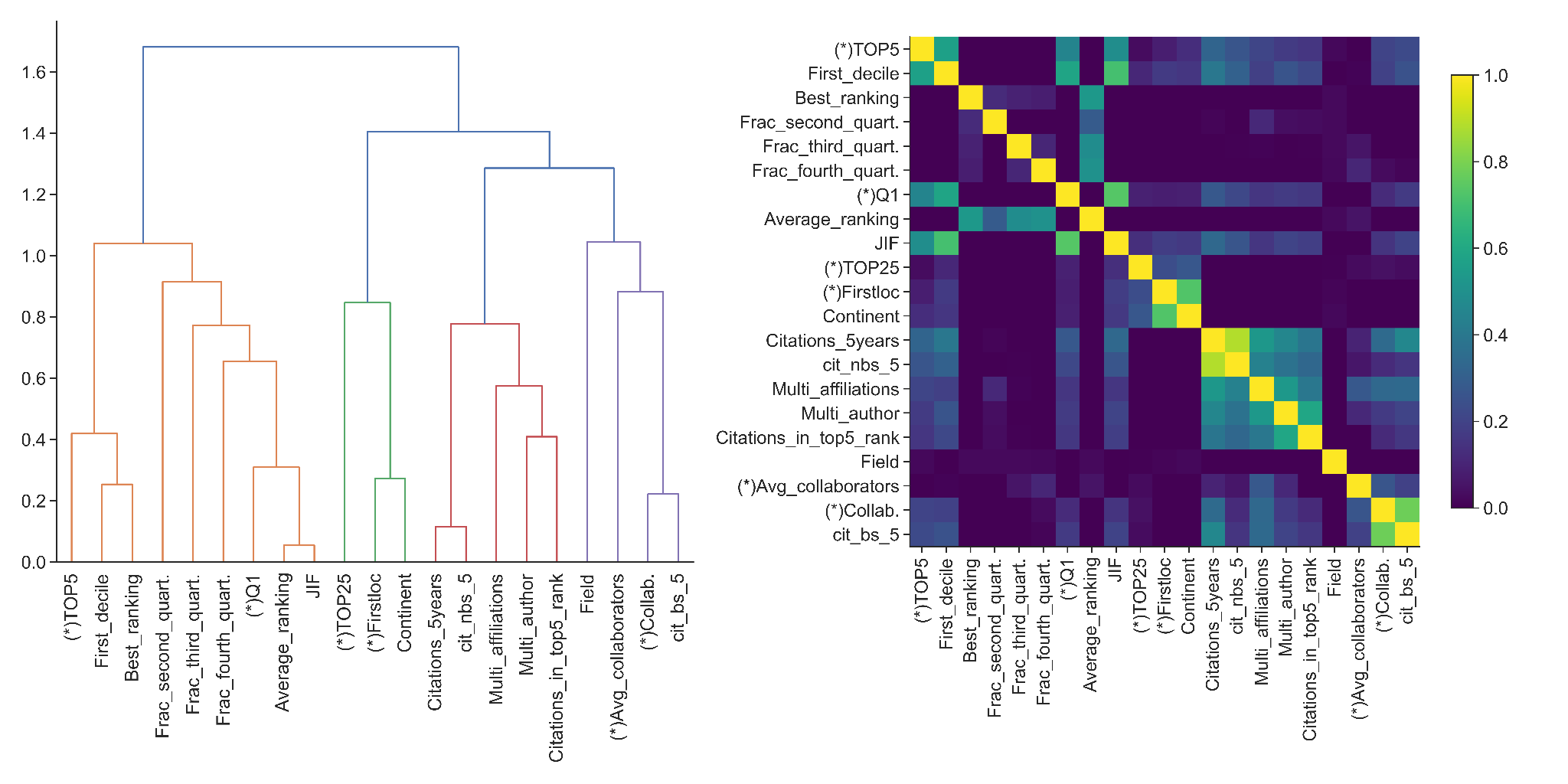


**Supplementary Figure 26. Dendrogram and correlation matrix for variables in Table 1: (**Left panel) Dendrogram of correlation for early-career factors collected (Table 1). Distances between variables have been calculated using Ward’s linkage (Ward J. H., JASA 58:301, 236-244, 1963). (Right panel) Correlation matrix. Each element shows the Spearman’s rank correlation between (row,column) pair of variables. Matrix elements are coloured according to the colorbar on the right hand side; the lighter the colour, the higher the correlation between a pair of variables. Variables marked with a (*) correspond to variables used in Models 1, 2 and 3 in the random forest classification analysis. Note how these variables are far away in the dendrogram in terms of distance (vertical axis, left panel) and correlation value (right panel).

***
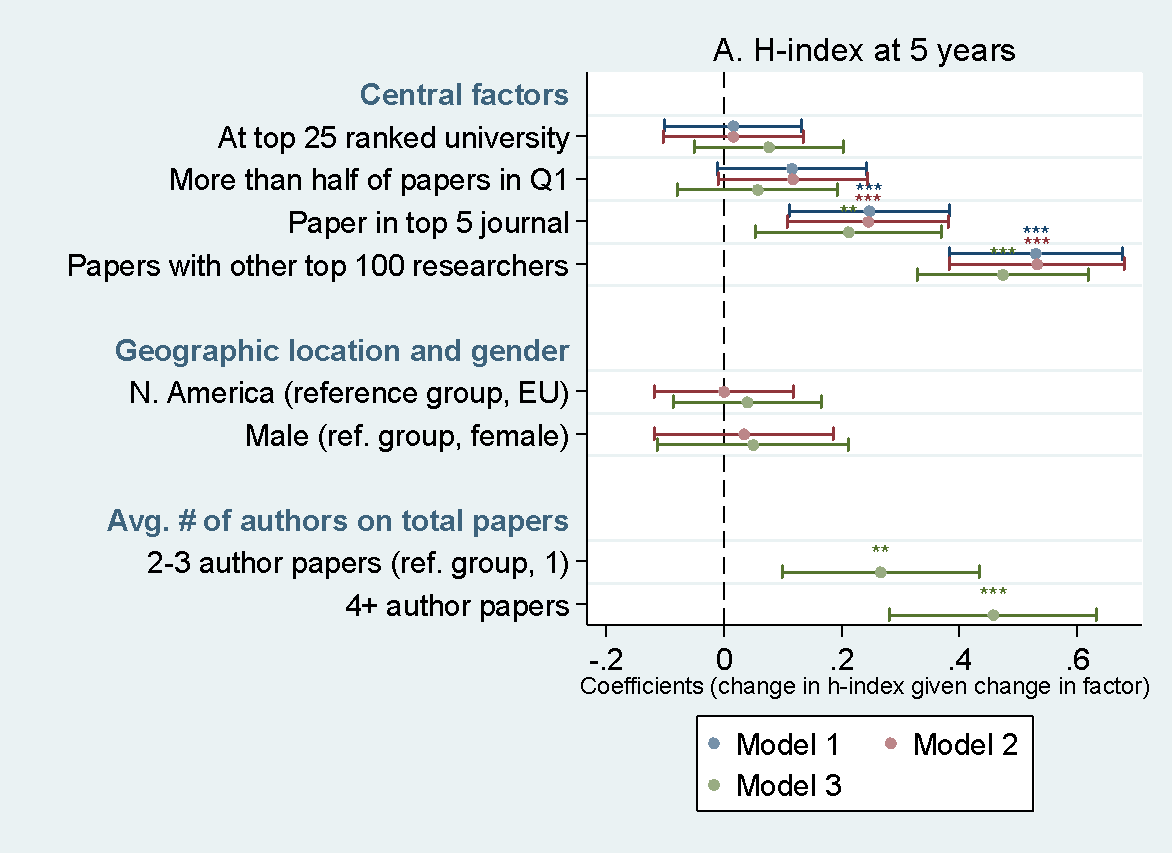

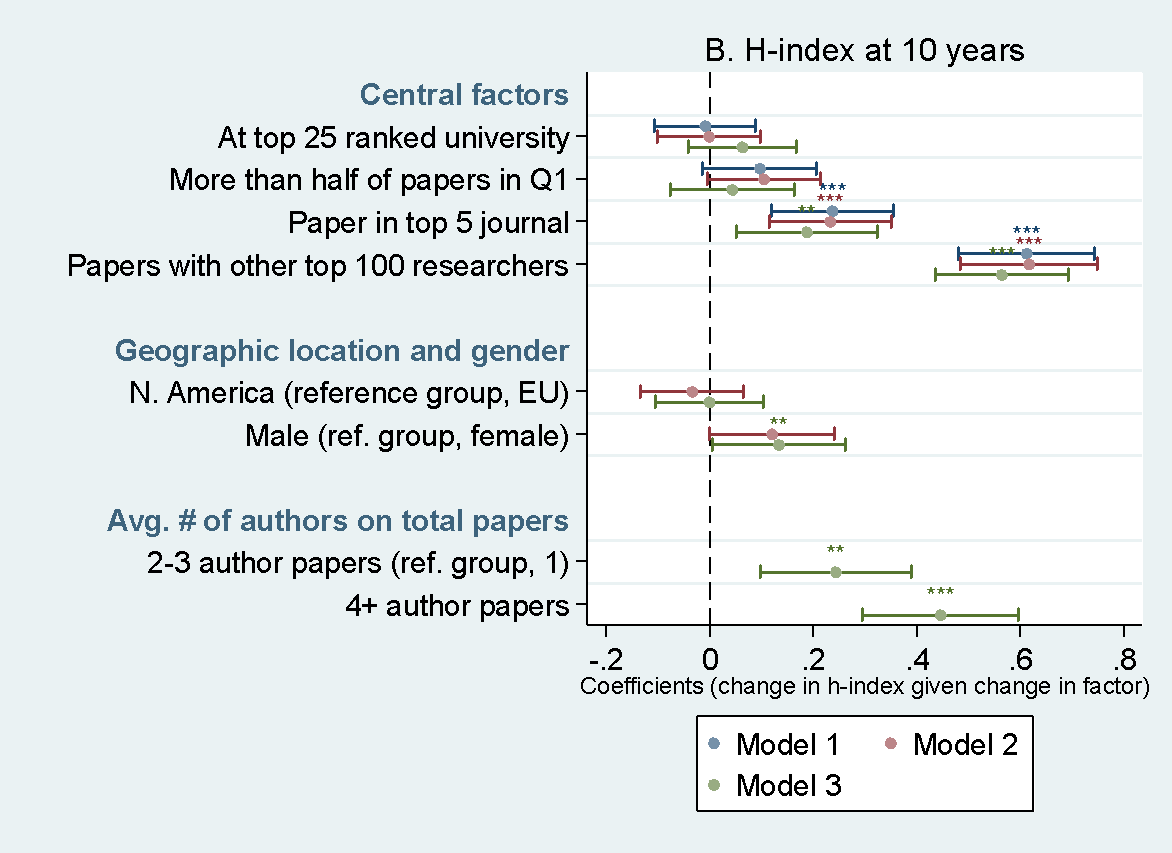
***

***
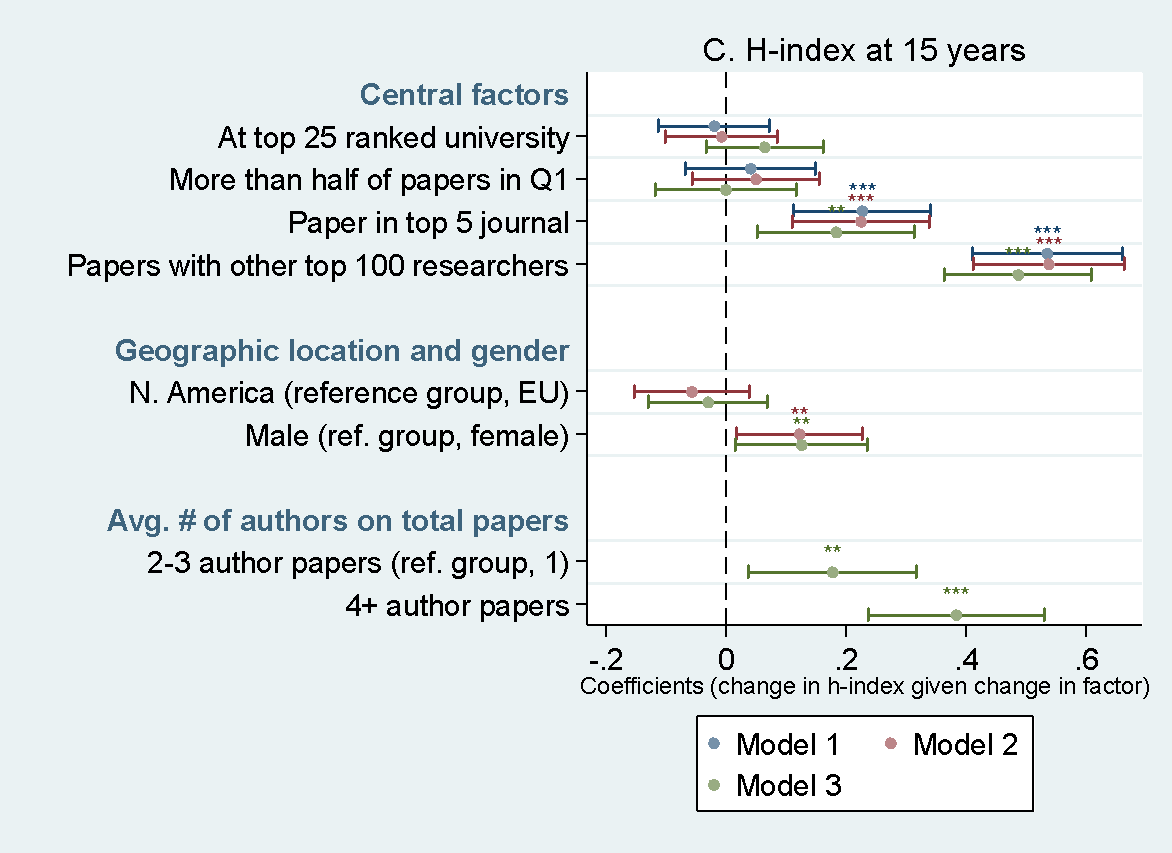

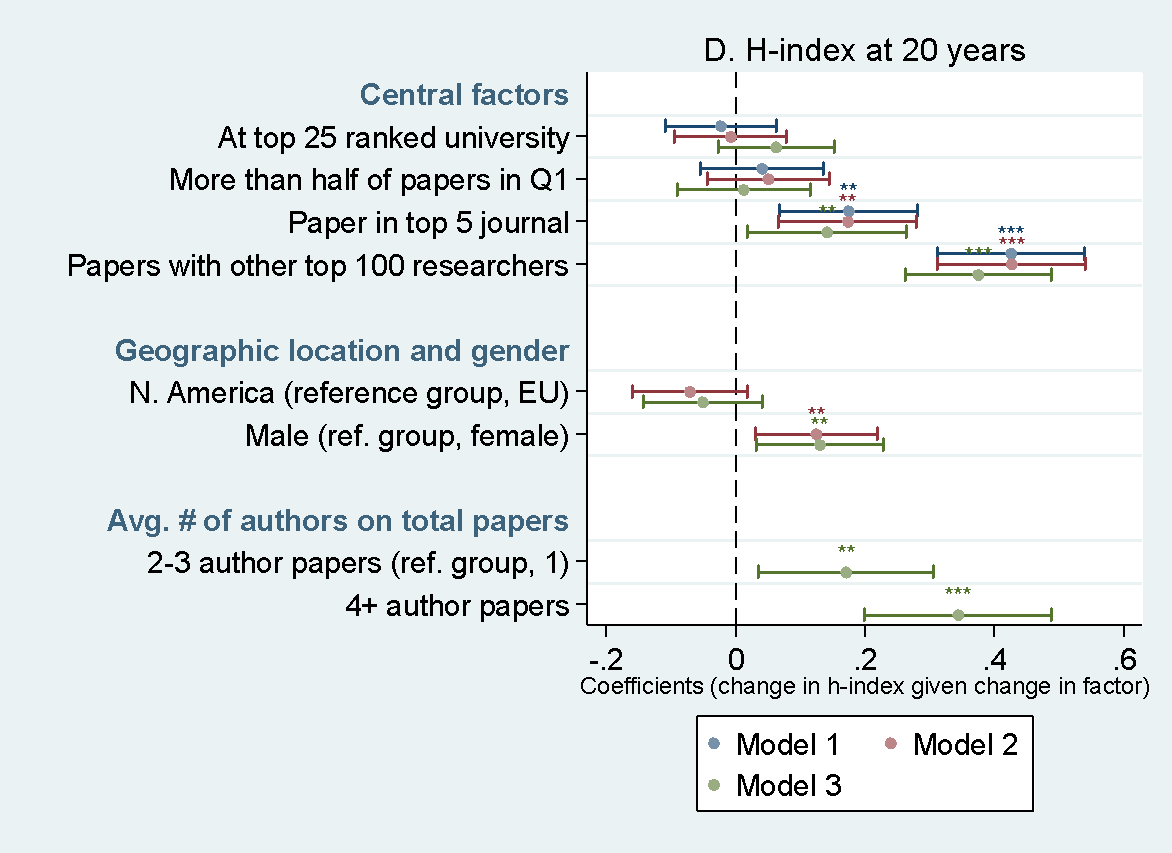
***

**Supplementary Figure S27. Regression results: Factors driving h-index in first 5, 10, 15 and 20 years for the world’s prominent researchers.** A. The R^2^ is 0.12, 0.12 and 0.15 for models 1-3 respectively. B. The R^2^ is 0.19, 0.19 and 0.22 for models 1-3 respectively. C. The R^2^ is 0.16, 0.16 and 0.19 for models 1-3 respectively. D. The R^2^ is 0.12, 0.13 and 0.16 for models 1-3 respectively. All independent variables reflect the first five years of researchers’ career. Statistical significance: *** <1%, ** <5%,* <10%. For the four key factors, the reference groups are defined as outlined in Supplementary Figure S25. The OLS regression analyses here illustrate that publishing in a top 5 journal within a given field and especially collaborating with other prominent researchers during the first five years are significant predictors of the h-index at 5 years, while holding geography, gender, number of co-authors and scientific fields constant (panel A). Again, we find that not only collaborating with prominent researchers but also larger collaborations in general that comprise of publishing in larger teams is also predictive of h-index at 5 years. Importantly, we find again that neither gender nor geographic location are predictors of differences in h-index 5 years after the start of a scientific career. Collaborating with other prominent researchers early on appears to thus be the strongest determinant of later prominence. We find the same predictors for h-index at 10, 15 and 20 years since the first publication (panel B, C and D) as we did for h-index at 5 years (panel A). The only difference we find is that gender becomes an explanatory variable for changes in h-index: male researchers experience about a 13% increase in h-index compared to females. This is consistent with findings that show that female faculty generally produce less research than male faculty and as a result have lower h-indices. ^(41)^


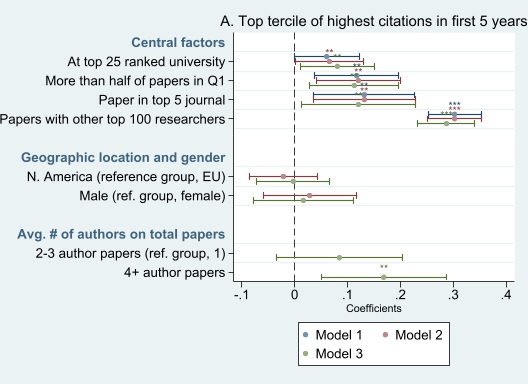

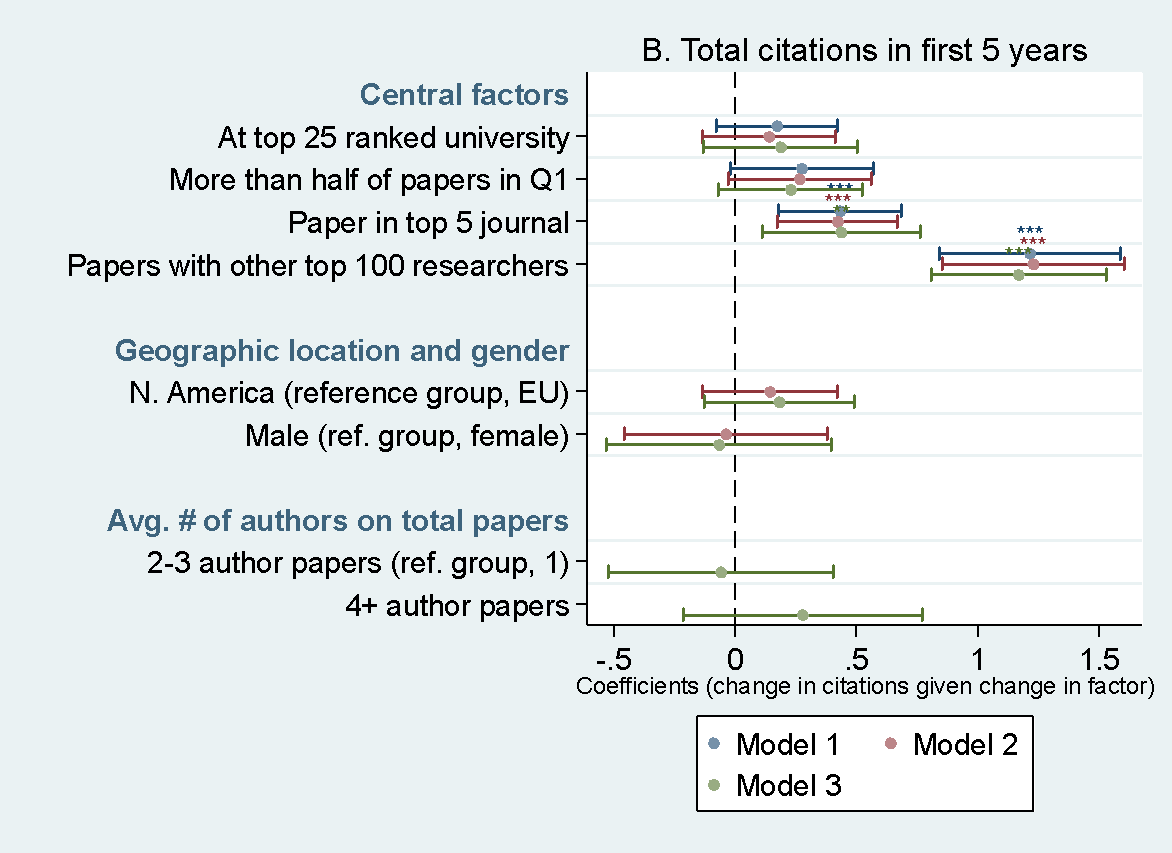


**Supplementary Figure S28. Regression results: Factors driving the highest third most-cited prominent researchers (A) and the total citations of prominent researchers (B) in first 5 years**. **A.** The R^2^ is 0.16, 0.17 and 0.17 for models 1-3 respectively. B. The R^2^ is 0.12, 0.13 and 0.13 for models 1-3 respectively. All independent variables reflect the first five years of researchers’ career. Statistical significance: *** <1%, ** <5%,* <10%. For the four key factors, the reference groups in the regressions are not being at a top 25 ranked university (with results thus illustrating the increase in citations for being at a top 25 university), not having published more than half of papers in Q1 journals, not having a paper published in a top 5 journal, and not having a paper published with other top 100 researchers. Using a logistic regression, we find that these key factors are significant predictors of reaching the highest citation tercile (panel A). Specifically, we find that in the first 5 years, the top third most cited researchers are about 8% more likely to have researched at a top 25 university, about 11% more likely to have published more than half of their papers in quartile 1 journals, about 12% more likely to have published a paper in a top 5 ranked journal in their field and about 29% more likely to have published papers with other prominent researchers, while controlling for geographic location, gender, number of co-authors and scientific fields. As with collaboration with prominent researchers, larger collaborations in general in which researchers publish in large teams can also be predictive of falling into the top citation tercile (panel A). **B.** the OLS regression illustrates that in these first 5 years, a researcher’s total citations increase by about 40% for those who published a paper in a top 5 ranked journal in their field (compared to those who did not) and by about 120% for those who published papers with other top 100 researchers (compared to those who did not), while controlling for geographic location, gender, multi-authorship and scientific fields (panel B). We thus find that all four factors are significant predictors for reaching the top of the top in citations (panel A) while having a top 5 paper and collaborating with prominent researchers are significant predictors of making it to the top 100 in general (panel B).


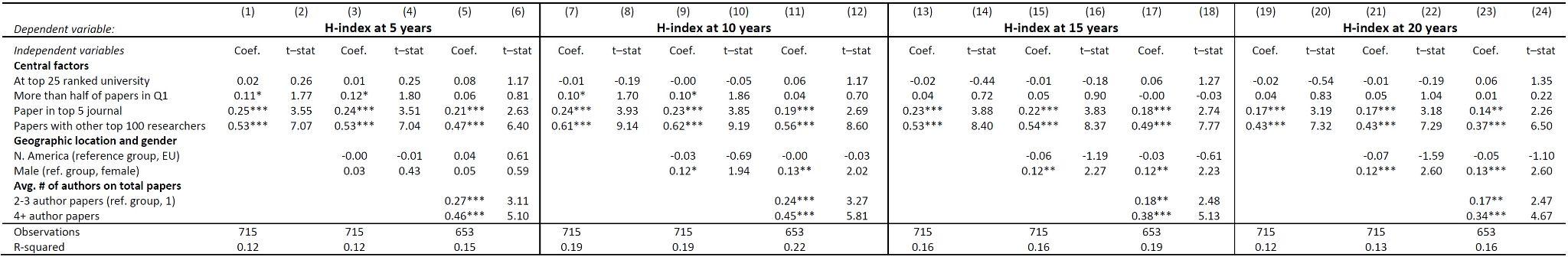


**Supplementary Table 1. Regression results: Factors driving h-index in first 5, 10, 15 and 20 years for the world’s prominent researchers.** Results reflect the same data in Supplementary Figure S27, but in table format – see that figure for greater details.
